# Supplementary material for: Dynamic heterogeneity in COVID-19: Insights from a mathematical model
Source: PLoS One. 2024 May 31;19(5):e0301780. doi: 10.1371/journal.pone.0301780 (PMC11142552; doi:10.1371/journal.pone.0301780)
Supplement: S1 Appendix — (DOCX) [file pone.0301780.s001.docx]

**Supplementary Information for**

Dynamic heterogeneity in COVID-19: Insights from a mathematical model

Chrysovalantis Voutouri^1,2,¶^, C. Corey Hardin^3, ¶^, Vivek Naranbhai^4,5,6^, Mohammad R. Nikmaneshi^1^, Melin J. Khandekar^7^, Justin F Gainor^4^, Lance L. Munn^1,*^, Rakesh K. Jain^1,*^ and Triantafyllos Stylianopoulos^2,*^

* Corresponding author

Triantafyllos Stylianopoulos, Lance L. Munn, Rakesh K. Jain

Email: tstylian@ucy.ac.cy, munn@steele.mgh.harvard.edu, rjain@mgh.harvard.edu

**This PDF file** **includes:**

Supplementary text

Figures S1 to S10

Tables S1 to S5

SI References

**Description of mathematical model**

The model consists of a series of differential equations which describe the dynamics of infection of epithelial cells in the lung by SARS-CoV-2, the innate immune response to infection, including the production of pro- and anti-inflammatory cytokines and the activation of the coagulation cascade. The model further accounts for interactions between the virus and immune cells including neutrophils, B cells and T cells.

**Viral infection**: SARS-CoV-2 enters the cell by docking to ACE2. ACE2 can be membrane-bound or soluble, and it regulates inflammation by converting Ang II to Ang 1-7 and Ang I to Ang 1-9 [1]. Intracellular virus initiates inflammatory pathways through toll-like receptors and NFκB, which produces interferons and other inflammatory cytokines. In the presence of inflammatory cytokines and virus, neutrophils proliferate and can produce neutrophil extracellular traps (NETs). Viral antigens and inflammatory cytokines further facilitate activation of naïve T cells, creating virus-specific T effector cells. T cell activation is controlled by viral antigen strength and the status of immune checkpoint inhibition (specifically PD-L1/PD-1).

**Coagulation cascade**. Viral infection and the resulting high levels of inflammatory cytokines in the plasma, can result in thrombosis. Damage to endothelial cells and the production of NETs can exacerbate the thrombosis, and microthrombi can travel through the blood circulation to accumulate in other organs, including the brain, heart and lung. We use a simplified model of the coagulation pathways, assuming that formation of microthrombi is proportional to the number of infected endothelial cells, the presence of neutrophil NETs, and level of inflammatory cytokines. Transport of oxygen from the alveolar space to the blood vessels in the lung is calculated using a modified diffusion model, which accounts for local thrombosis and tissue damage via an increase in the resistance to diffusion of oxygen.

**Vaccination-induced immunity**. We explicitly model the separate mechanisms of mRNA and vector vaccines. The vaccines, as particles, either lipid nanoparticles in the case of mRNA vaccines or viral-vector in the case of vector vaccines, enter host cells and either induce DNA transcription to mRNA (vector vaccine) and then translation into viral antigen or result directly in translation of viral antigens (mRNA vaccine). Subsequently, vaccine-induced peptides exit the cells and interact with dendritic cells to produce antigen presenting cells. These subsequently activate T cells and B cells to create CD4+ and CD8+ effector and memory T cells as well as short-lived and long-lived plasma (antibody-secreting) B cells.

**Pharmacokinetic-pharmacodynamic (PK/PD) model**. A PK/PD model has been formulated to allow for transport of viral particles, antibodies, cytokines and micro-thrombi among major compartments of the body, including the: lung, heart, liver, brain, spleen, gastro-intestinal, upper body, lower body, torso, and cardiac vessels (**Supplementary** **Figure 1**).

**Supplementary Figures and Tables**

**Supplementary Figure 1.** PK/PD model of COVID-19 infection and thrombosis simulates events that happen throughout the body when a patient contracts COVID-19. Virus exits the lung via the systemic circulation, and can infect endothelial cells (ECs) in various organs, modeled here as well-mixed compartments. Upon infection, viral replication and cell death cause vessel damage and local inflammation that induce thrombosis. Each tissue can then become a source of micro-thrombi, which enter the systemic circulation and can accumulate in the microvessels of the heart, lung and brain, inducing ischemic events.

**
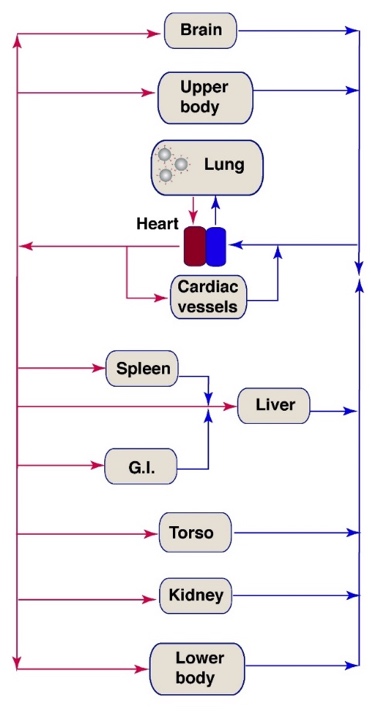
**

**Supplementary Figure 2.** Model comparison with clinical data of neutralization for the ancestral and the omicron variants. Clinical data of neutralization essays were employed to fit the model and determine the degradation rate of free virus by antibodies produced by vaccination. The experiments were simulated having the virus to be killed only by antibodies produced after a first booster dose. In the plot, the kite scatter symbols correspond to clinical data [2-5] and the circle to model predictions. Error bars present the standard error of antibody concentration for the range of values of model parameters considered (Supplementary Table 1). The table presents the values of the model parameter for the degradation rate of the free virus by the antibodies found by the fitting of the model to the experimental data. The values are normalized to the baseline value of the ancestral


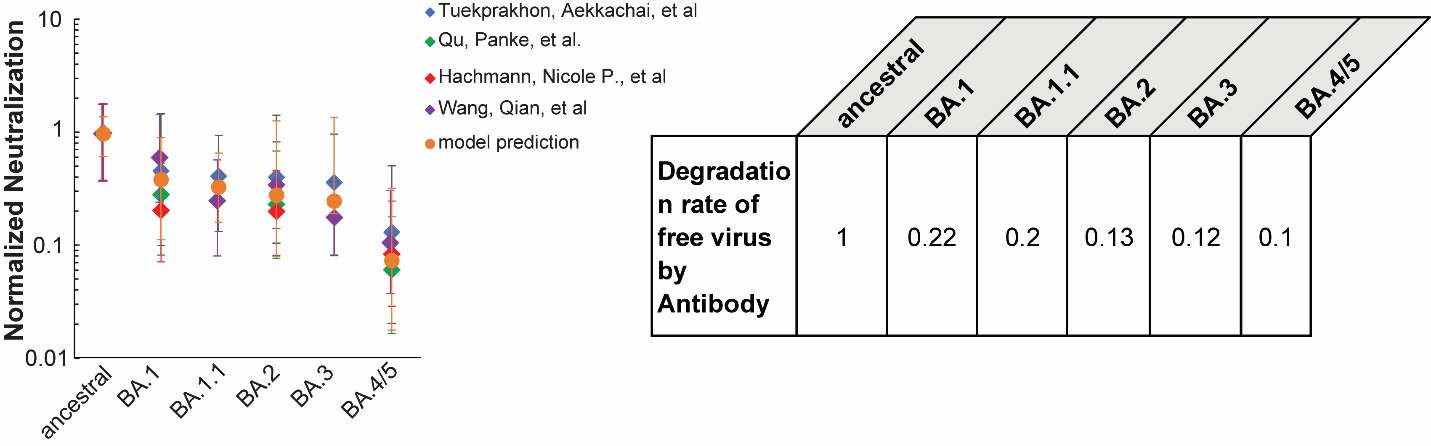


**Supplementary Figure 3.** Model predictions and validation with clinical data of Proportion Recovered for anti-viral treatment. The blue and orange lines correspond to clinical data [6-8], and the grey and yellow lines to model predictions. The sum of the squared error (i.e., χ2 value) between the clinical data for each of the studies and the corresponding model predictions for the baseline values of model parameters were calculated. For the temporal variation of antibody levels in healthy: χ2=0.8745. Error bars present the standard error for all values of model parameters considered. χ2 is the sum of the squared difference between the clinically measured, Pclin, and the predicted by the model, Pmodel, Proportion Recovered divided by the number of clinical data n, $\chi^{2}=\frac{1}{n}\sum_{i=1}^{n} \left[ \left( {Ct}_{clin}-{Ct}_{clin} \right)_{i}^{2} \right]$.


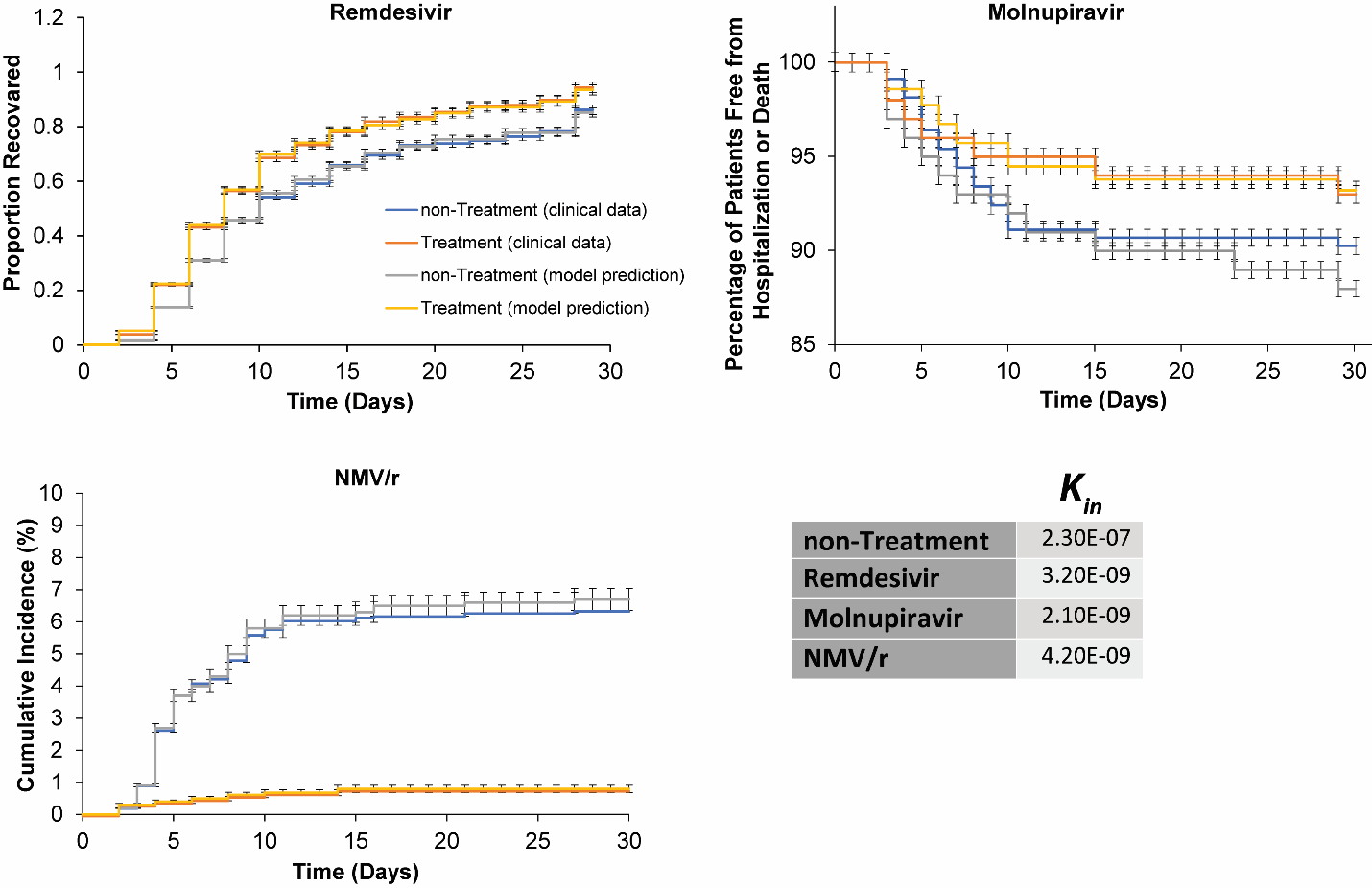


**Supplementary Figure 4.** Heterogeneity and hyperinflammation, hypercoagulation and antiviral therapy. Phase diagrams of peak viral load for small and large increases in both micro-thrombus and cytokine production as a function of the relative decrease of virus replication, Kin, with antiviral drugs and the omicron variants. Viral load is normalized by division with the initial value 4.96x10^4^ [pg/ml]


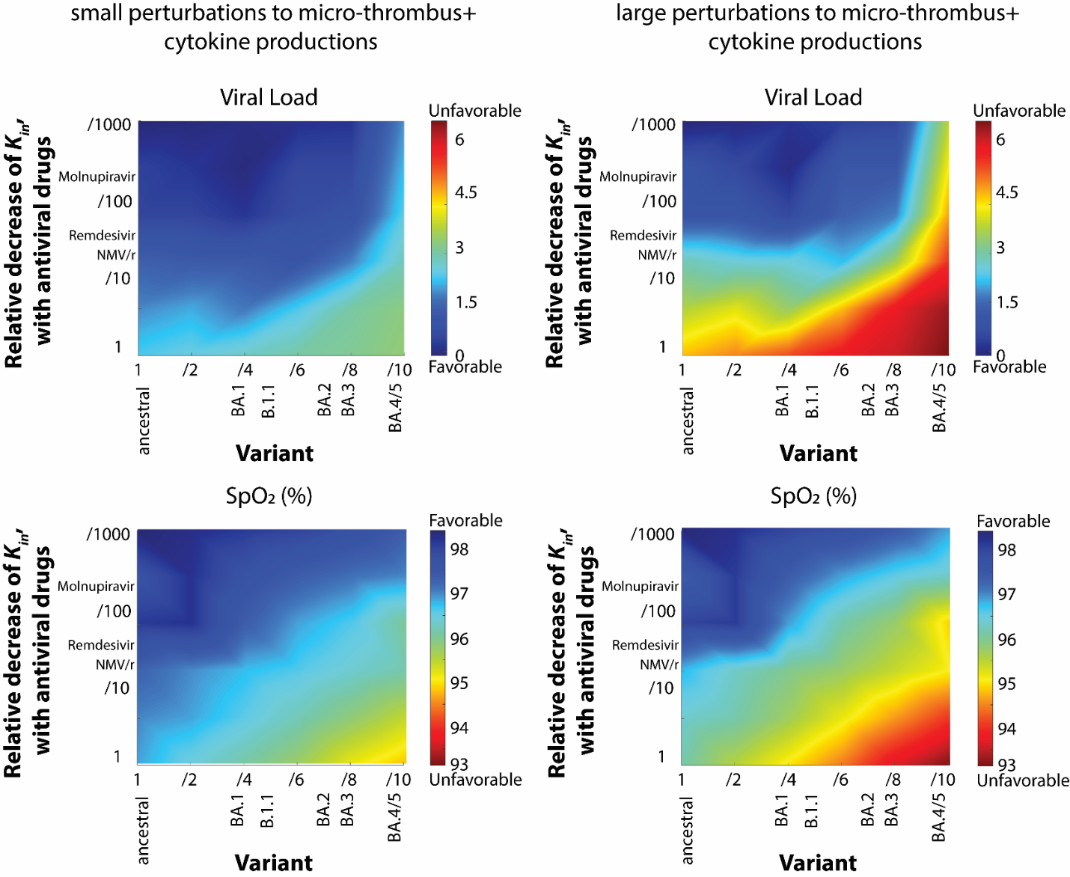


**Supplementary Figure 5**. **Hyperinflammation and innate immune effector cells** (A) Phase diagrams of macrophages and neutrophils as a function of the production rate of pro-inflammatory cytokines and a decrease in virus replication, K_in_, with antiviral treatment. (B) Phase diagrams of SpO_2_, microthrombus and viral load as a function of the production rate of pro-inflammatory cytokines and a decrease in virus replication, K_in_, with antiviral treatment and in the absence of neutrophils. Viral load, neutrophil levels and macrophage levels are normalized with respect to their initial values 4.96x10^4^ [pg/ml], 4.2x10^9^ [1/L] and 2.17x10^9^ [1/L], respectively


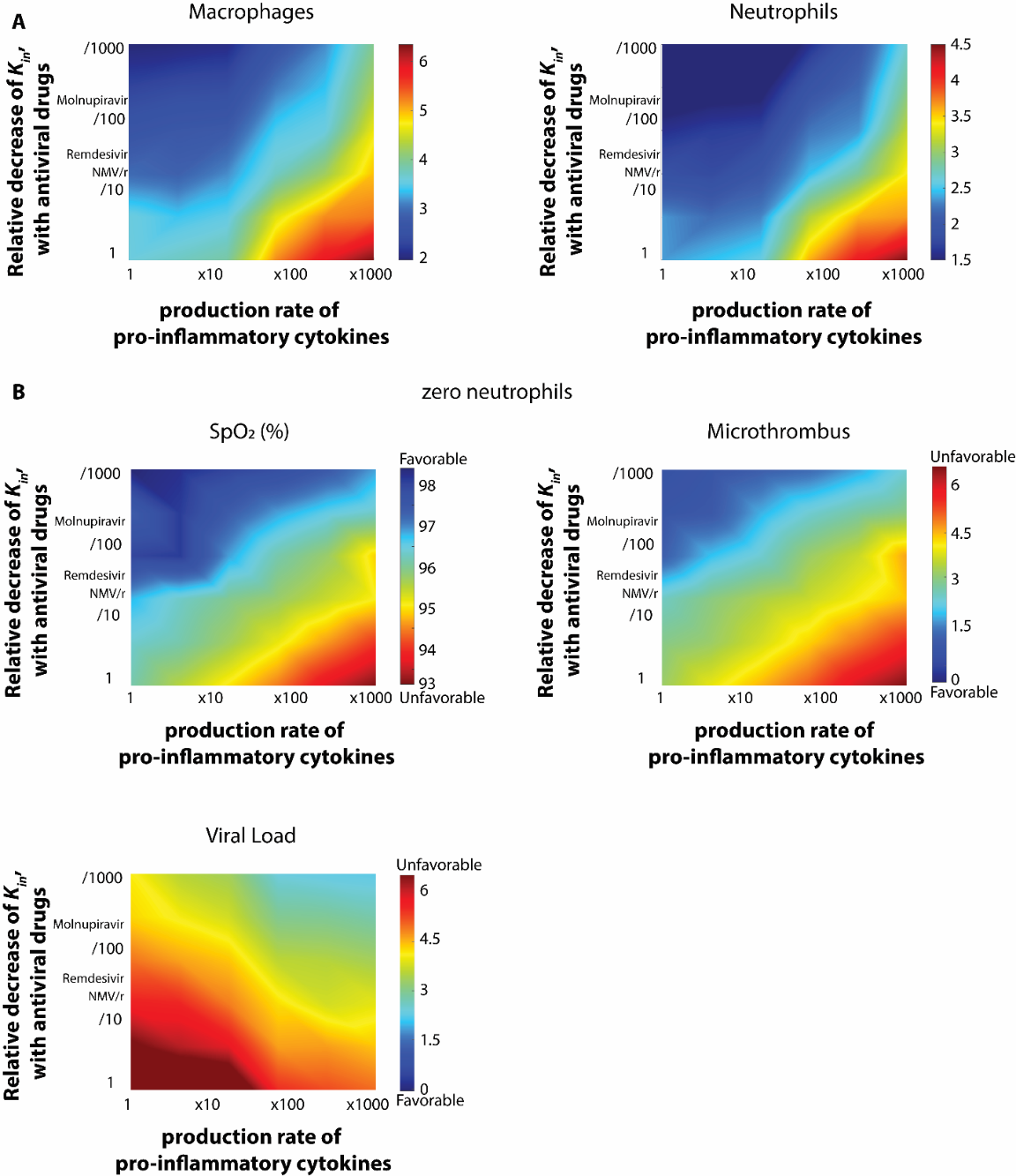


**Supplementary Figure 6**. Phase diagrams of peak viral load for small and large perturbations to production of naïve CD8^+^, CD4^+^ and B cells as a function of the relative decrease of virus replication, K_in_, with antiviral drugs and the omicron variants. Viral load is normalized by division with the initial value 4.96x10^4^ [pg/ml]


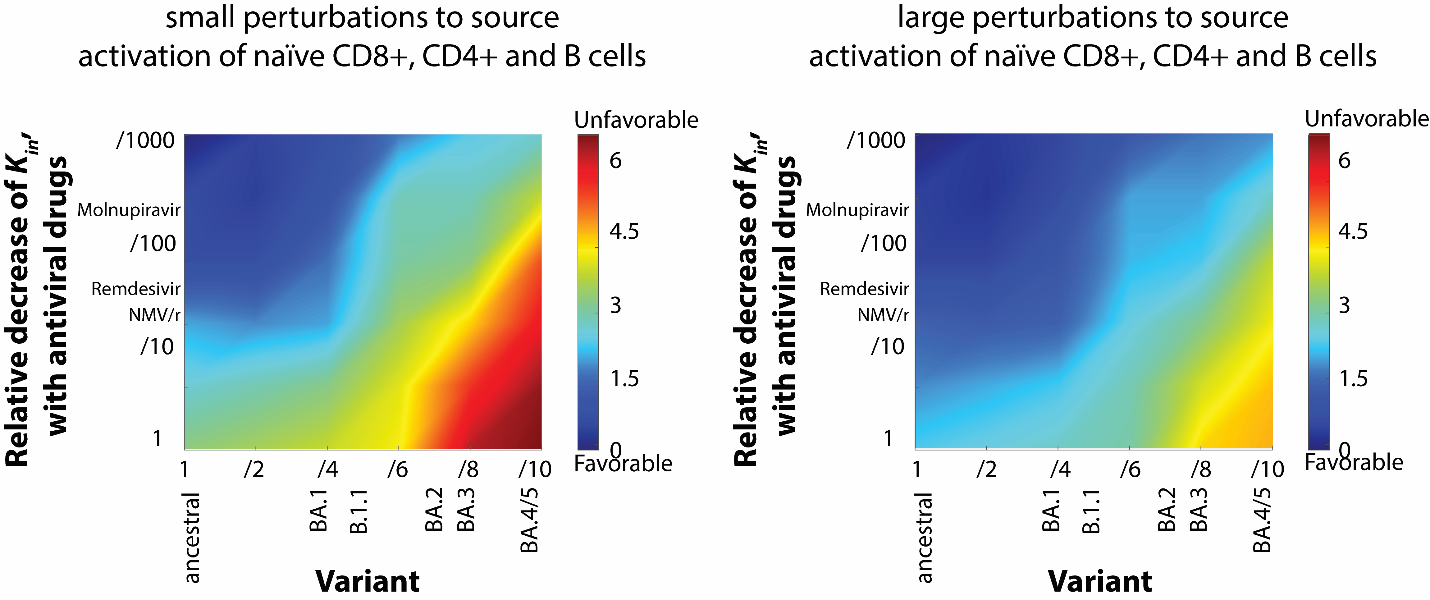


**Supplementary Figure 7**. **Interaction of initial viral inoculum and perturbations to the innate and adaptive immune response**. (A) Viral load over time in an untreated, vaccinated patient infected with antibody response consistent with the BA.4/5 variant. Increasing the expression of pro-inflammatory cytokines decreases peak viral load but this effect can be attenuated with a high initial inoculum of virus. Increasing viral load is associated with worsened gas exchange. Increasing the viral inoculum also attenuates the beneficial effect of a more robust T cell response (B).


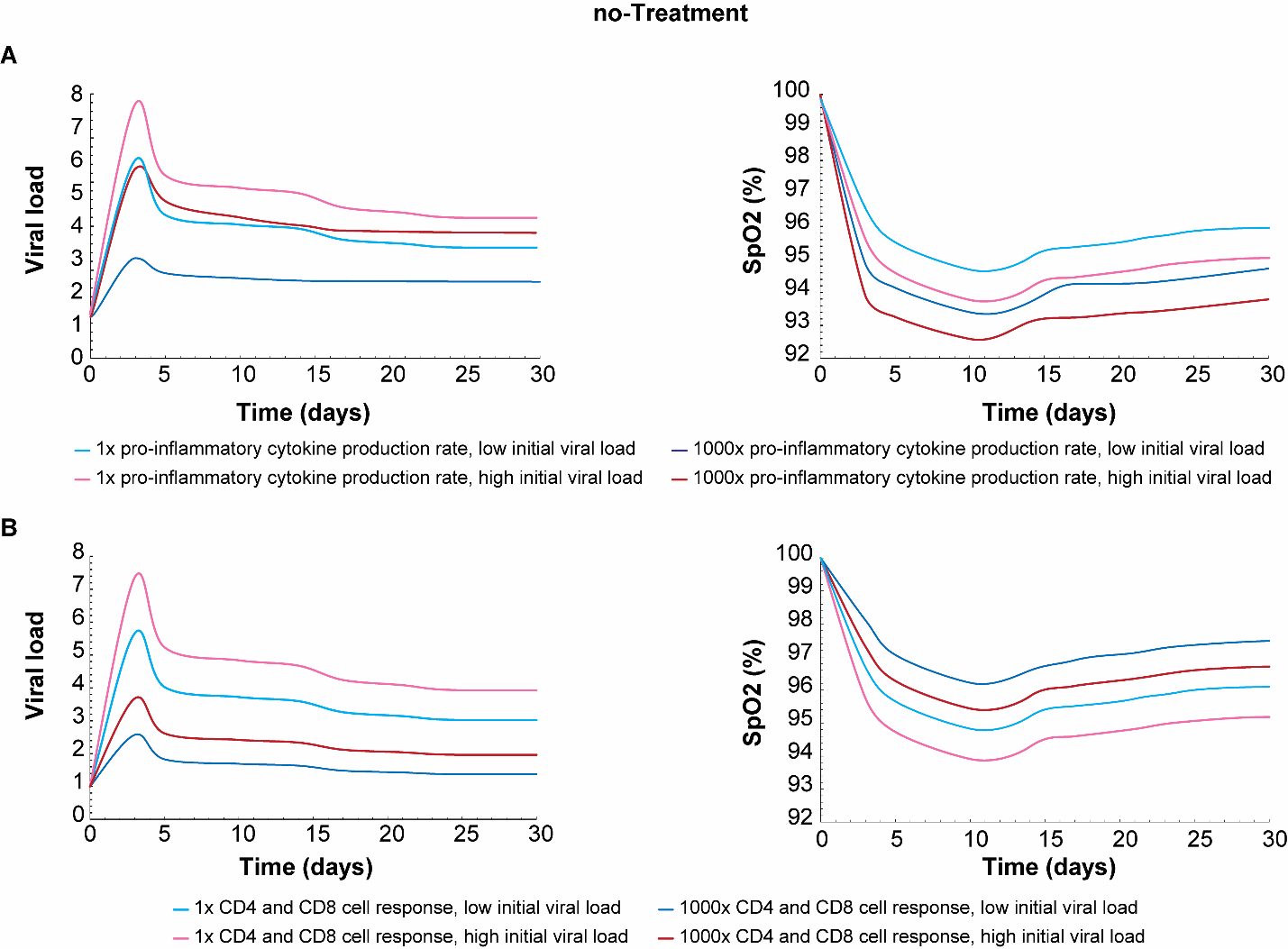


**Supplementary Figure 8**. **Time of treatment initiation affects disease rebound and therapeutic outcome.** (A) Temporal variation in levels of infected host cells, (B) oxygen saturation, SpO_2_, (C) Microthrombosis in the lung and (D) levels of memory CD8^+^ T cells for low risk (young) vaccinated patient and a low risk (young) unvaccinated patient induced by NMV/r as a function of treatment initiation. Antiviral treatments lasted for 10 days. Infected host cells and activated CD8^+^ T cells are normalized by division with value 14.46x10^6^ [1/ml] and 0.4 [g/cm^3^] respectively


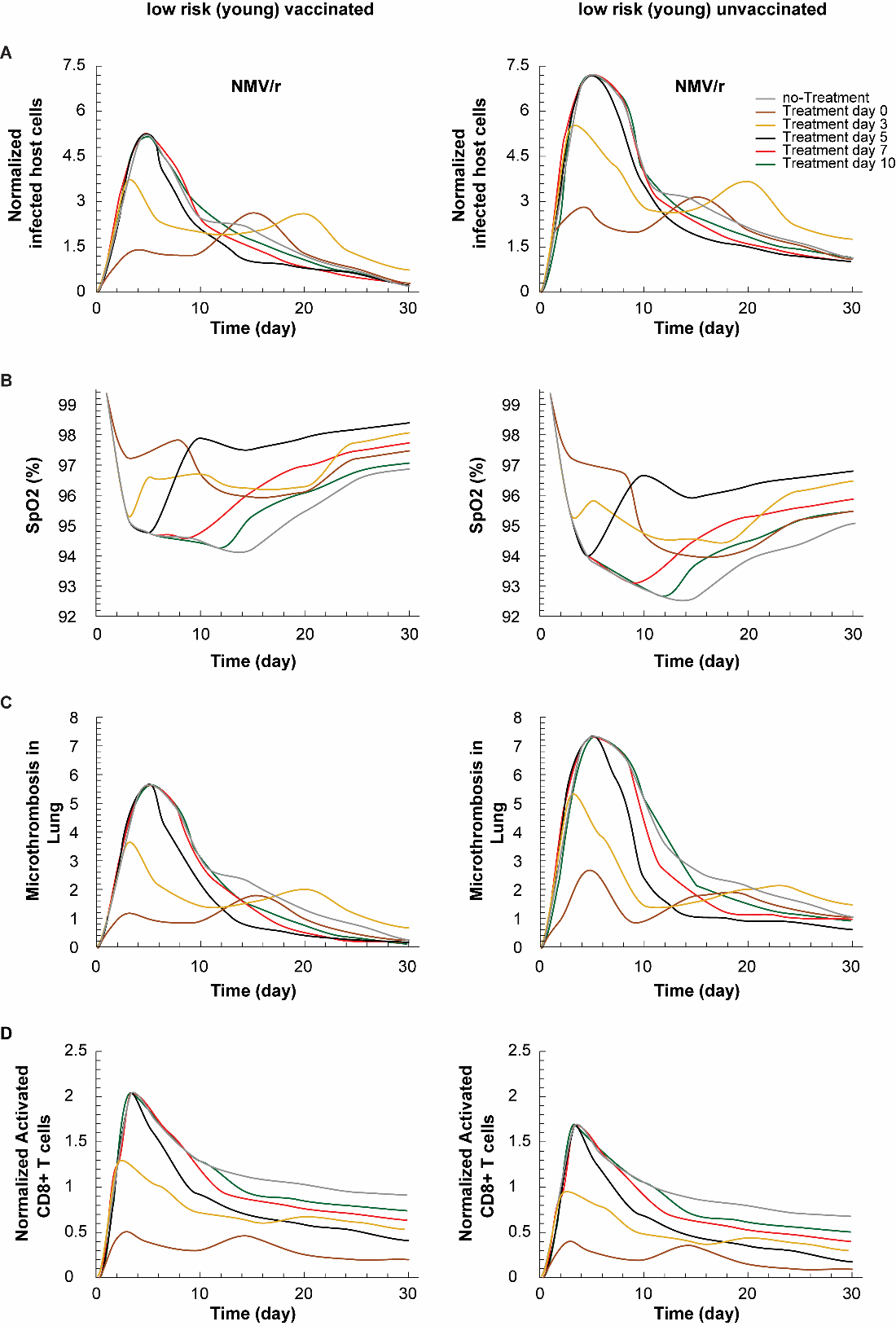


**Supplementary Figure 9**. **Time of treatment initiation affects disease rebound and therapeutic outcome.** (A) Temporal variation in levels of infected host cells, (B) oxygen saturation, SpO_2_, (C) Microthrombosis in the lung and (D) levels of memory CD8^+^ T cells for three patient phenotypes induced by remdesivir as a function of treatment initiation. Antiviral treatments lasted for 10 days. Infected host cells and activated CD8^+^ T cells are normalized by division with value 14.46x10^6^ [1/ml] and 0.4 [g/cm^3^] respectively.


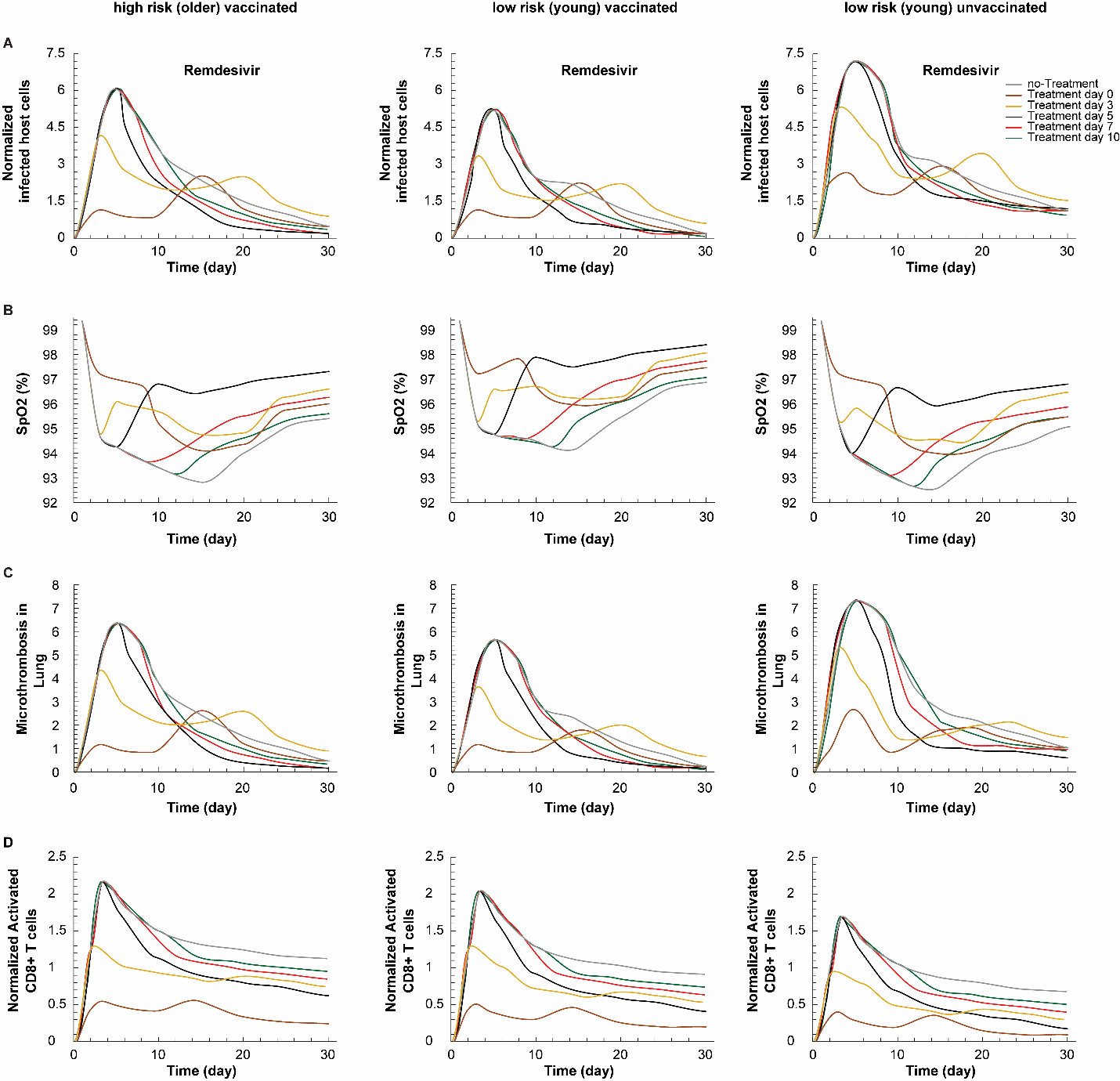


**Supplementary Figure 10**. **Time of treatment initiation affects disease rebound and therapeutic outcome.** (A) Temporal variation in levels of infected host cells, (B) oxygen saturation, SpO_2_, (C) Microthrombosis in the lung and (D) levels of memory CD8^+^ T cells for three patient phenotypes induced by Molnupiravir as a function of treatment initiation. Antiviral treatments lasted for 10 days. Infected host cells and activated CD8^+^ T cells are normalized by division with value 14.46x10^6^ [1/ml] and 0.4 [g/cm^3^] respectively.


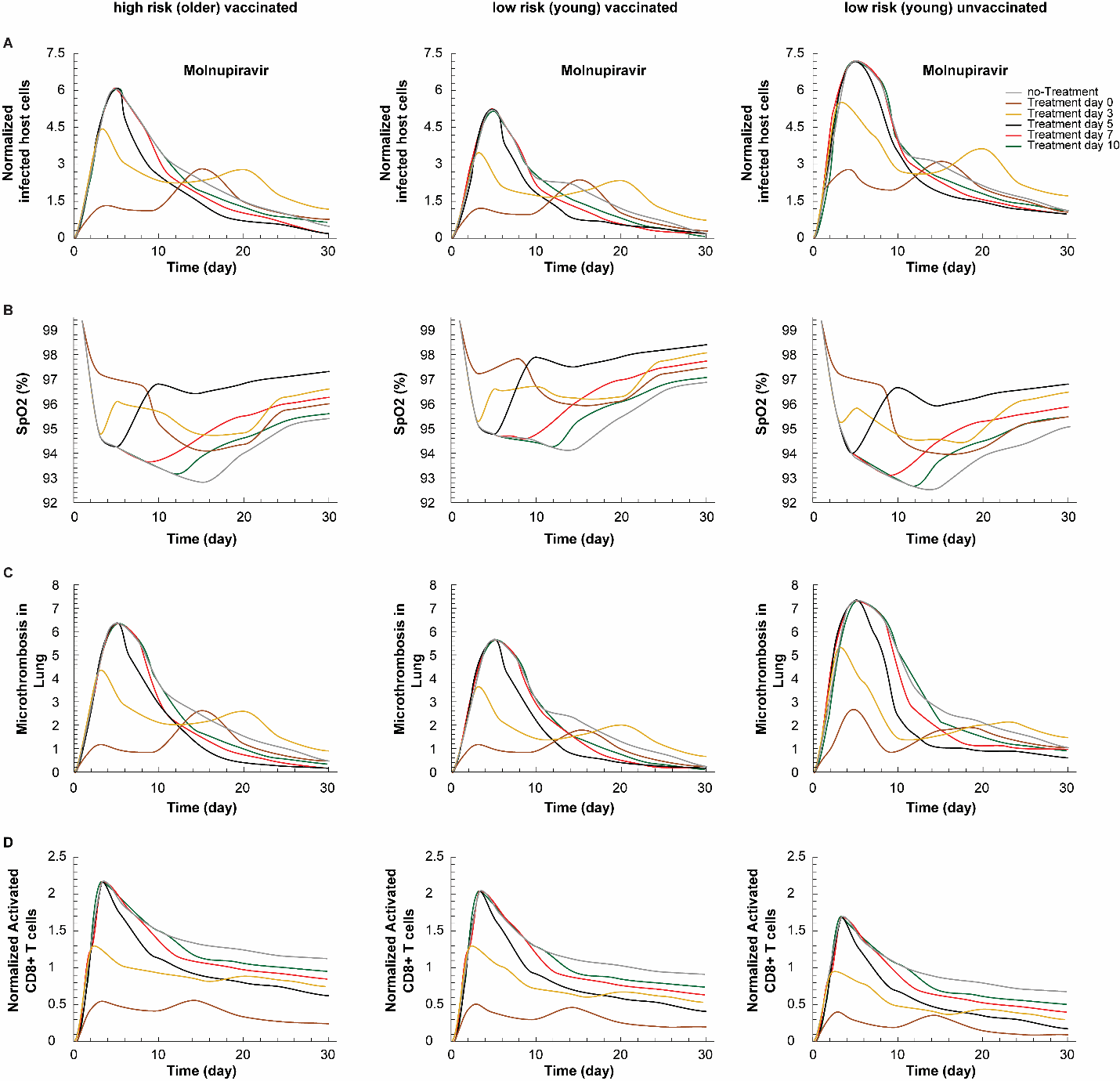


**Supplementary Table 1**: Values of Model parameters.

| Parameter | Description | Value [Units] | Reference |
| --- | --- | --- | --- |
| *K_AGT_* | Angiotensinogen production rate | 2.27x10^6^ [nmol/L/h] | Pilvankar et al. [9] |
| *c_Renin_* | Renin rate constant | 1.8x10^-14^ [1/s] | Pilvankar et al. [10] |
| *h_AGT_* | Angiotensinogen half-life | 10 [h] | Pilvankar et al. [9] |
| $\boldsymbol{h}_{\boldsymbol{Renin}}$ | Renin half-life | 0.25 [h] | Pilvankar et al. [9] |
| $\boldsymbol{K}_{\boldsymbol{f}}$ | Parameter for ANGII-renin feedback | 4.91x10^-5^ [1/h] | Pilvankar et al. [9] |
| *f* | Parameter for ANGII-renin feedback | 0.51 [nmol/ml] | Pilvankar et al. [9] |
| $\boldsymbol{K}_{\boldsymbol{Renin}}$ | Ang I production rate constant | 6.44x10^4^ [1/h] | Pilvankar et al. [9] |
| $\boldsymbol{K}_{\boldsymbol{ACE}}$ | Rate of conversion of ANGI->ANGII | 185.22[1/h] | Pilvankar et al. [9] |
| $\boldsymbol{K}_{\boldsymbol{NEP}}$ | Rate of conversion of ANGI->ANG(1-7) | 0.583 [1/h] | Pilvankar et al. [9] |
| $\boldsymbol{h}_{\boldsymbol{ANGI}}$ | Half-life of ANGI | 1.72x10^-4^ [h] | Pilvankar et al. [9] |
| $\boldsymbol{K}_{\boldsymbol{APA}}$ | Rate of conversion of ANGII->ANGIII | 43.6 [1/h] | Pilvankar et al. [9] |
| $\boldsymbol{h}_{\boldsymbol{ANGII}}$ | Half-life of ANGII | 5x10^-3^ [h] | Pilvankar et al. [9] |
| $\boldsymbol{h}_{\boldsymbol{AT}\boldsymbol{1}\boldsymbol{R-ANGII}}$ | Half-life of AT1R bound to ANGII | 1.5 [min] | Pilvankar et al. [10] |
| $\boldsymbol{h}_{\boldsymbol{AT}\boldsymbol{2}\boldsymbol{R-ANGII}}$ | Half-life of AT2R bound to ANGII | 1.5 [min] | Pilvankar et al. [10] |
| $\boldsymbol{h}_{\boldsymbol{ANG}_{\boldsymbol{(1-7)}}}$ | Half-life of ANG(1-7) | 30 [min] | Pilvankar et al. [10] |
| $\boldsymbol{h}_{\boldsymbol{MAsR-}\boldsymbol{ANG}_{\boldsymbol{(1-7)}}}$ | Half-life of Ang(1-7) bound to MAsR | 1.5 [min] | From model validation – same as *h _(AT1R-ANGII)_* |
| $\boldsymbol{h}_{\boldsymbol{ANG}_{\boldsymbol{(1-9)}}}$ | Half-life of ANG(1-9) | 24 [min] | Pilvankar et al. [10] |
| $\boldsymbol{K}_{\boldsymbol{APM}}$ | Rate of conversion of ANGIII->ANGIV | 43.6 [1/h] | From model validation – same as K_APA_ |
| $\boldsymbol{h}_{\boldsymbol{ANGIII}}$ | Half-life of ANGIII | 30 [s] | Pilvankar et al. [10] |
| $\boldsymbol{h}_{\boldsymbol{ANGIV}}$ | Half-life of ANGIV | 0.5 [min] | Lo A. et al. [11] |
| $\boldsymbol{h}_{\boldsymbol{AT}\boldsymbol{4}\boldsymbol{R-ANGIV}}$ | Half-life for ANGIV bound to AT4R | 1.5 [min] | From model validation – same as *h _(AT1R-ANGII)_* |
| $\boldsymbol{D}$ | Diffusion of the virus in the lung | 5x10^-3^ [cm^2^/s] | From model validation – Mok W. et al. [12] |
| $\boldsymbol{K}_{\boldsymbol{d}}$ | Inactivating rate of the virus | 4.8x10^-5^ [1/s] | Mok W. et al. [12] |
| $\boldsymbol{K}_{\boldsymbol{a}}$ | Rate of virus replication and release from the cell | 5.78x10^2^ [1/s] | From model validation - Mok W. et al. [12] |
| *K_IF_* | Strength of virus replication inhibition by Interferon | 0.0025[pg/ml] | From model validation |
| $\boldsymbol{K}_{\boldsymbol{int}}$ | Rate of bound virus internalization | 5.78x10^-4^ [1/s] | Mok W. et al. [12] |
| $\boldsymbol{K}_{\boldsymbol{AT}\boldsymbol{1}\boldsymbol{R}}$ | Rate of production of pro-inflammatory cytokines | 4.2 x10^2^ [pg/h/fmol] | From model validation |
| $\boldsymbol{S}_{\boldsymbol{n}}$ | Cytokine production by innate immune cells and infected cells | 2.1x10^-2^ [pg/h] | Smith A.M. et al [13] |
| $\boldsymbol{S}_{\boldsymbol{c}}$ | Cytokine production by the internalized virus | 2.9 x10^-2^ [pg/ml/h] | Smith A.M. et al [13] |
| $\boldsymbol{d}_{\boldsymbol{c}}$ | Degradation rate of pro-inflammatory cytokines | 8.3x10^-1^ [1/h] | Smith A.M. et al [13] |
| $\boldsymbol{K}_{\boldsymbol{g}}$ | Production of anti-inflammatory cytokines by macrophages and neutrophils | 2.1x10^-2^ [pg/h] | Dunster J.L. et al. [14] |
| $\boldsymbol{\varphi}_{\boldsymbol{a}}$ | Production of anti-inflammatory cytokines by macrophages interaction neutrophils | 2.1x10^-6^ [ml] | Dunster J.L. et al. [14] |
| $\boldsymbol{K}_{\boldsymbol{Ang}\boldsymbol{1-7}}$ | Production rate of anti-inflammatory cytokines by ANG(1-7) bound to MAs receptor | 4.2x10^2^ [pg/h/fmol] | From model validation |
| $\boldsymbol{\gamma}_{\boldsymbol{a}}$ | Degradation rate of anti-inflammatory cytokines | 3 [1/day] | Dunster J.L. et al. [14] |
| $k_{H}^{b}$ | Rate of conversion of healthy to infected epithelial cells | 1.36x10^5^ [1/M/s] | Su Z. and Wu Y. [15] |
|  | Production of infected epithelial cells by neutrophils and pro/anti-inflammatory cytokines | 2.3x10^-9^ [ml/h] | From model validation |
| $\boldsymbol{R}_{\boldsymbol{H}}$ | Rate of production of healthy epithelial cells | 2.75x10^-3^ [1/h] | Mahasa KJ et al.[16] |
| $\boldsymbol{\chi}_{\boldsymbol{n}}$ | Production rate of neutrophils by pro-inflammatory cytokines | 2.1x10^-2^ [1/h] | From model validation- Dunster J.L. et al. [14] |
| $\boldsymbol{\gamma}_{\boldsymbol{n}}$ | Rate of production of neutrophil NETs | 6.3x10^-4^ [1/h] | Dunster J.L. et al. [14] |
| $\boldsymbol{\gamma}_{\boldsymbol{NETS}}$ | Degradation rate of NETs | 6.3x10^-7^[1/h] | From model validation |
| $\boldsymbol{\chi}_{\boldsymbol{m}}$ | Production rate of macrophages by pro-inflammatory cytokines | 0.02 [1/pg/h] | From model validation- Dunster J.L. et al. [14] |
| $\boldsymbol{\gamma}_{\boldsymbol{Ma}}$ | Degradation rate of macrophages | 6.3x10^-4^ [1/h] | Dunster J.L. et al. [14] |
| $\boldsymbol{K}_{\boldsymbol{ACE}\boldsymbol{2-ANGI}}^{\boldsymbol{on}}$ | Rate of ANGI binding to ACE2 receptors | 25 [ml/h/nmol] | From model validation – Mok W. et al. [12] |
| $\boldsymbol{K}_{\boldsymbol{ACE}\boldsymbol{2-ANGI}}^{\boldsymbol{off}}$ | Rate of ANGI detachment from ACE2 receptors | 5.22[1/h] | From model validation – Mok W. et al. [12] |
| $\boldsymbol{K}_{\boldsymbol{AT}\boldsymbol{1}}^{\boldsymbol{on}}$ | Rate of ANGII binding to AT1R receptors | 25 [ml/h/nmol] | From model validation – Mok W. et al. [12] |
| $\boldsymbol{K}_{\boldsymbol{AT}\boldsymbol{1}}^{\boldsymbol{off}}$ | Rate of ANGII detachment from AT1R receptors | 5.22[1/h] | From model validation – Mok W. et al. [12] |
| $\boldsymbol{K}_{\boldsymbol{AT}\boldsymbol{2}}^{\boldsymbol{on}}$ | Rate of ANGII binding to AT2R receptors | 25 [ml/h/nmol] | From model validation – Mok W. et al. [12] |
| $\boldsymbol{K}_{\boldsymbol{AT}\boldsymbol{2}}^{\boldsymbol{off}}$ | Rate of ANGII detachment from AT2R receptors | 5.22[1/h] | From model validation – Mok W. et al. [12] |
| $\boldsymbol{K}_{\boldsymbol{ACE}\boldsymbol{2-ANGII}}^{\boldsymbol{on}}$ | Rate of ANGII binding to ACE2 receptors | 25 [ml/h/nmol] | From model validation – Mok W. et al. [12] |
| $\boldsymbol{K}_{\boldsymbol{ACE}\boldsymbol{2-ANGII}}^{\boldsymbol{off}}$ | Rate of ANGII detachment from ACE2 receptors | 15.66[1/h] | From model validation – Mok W. et al. [12] |
| $\boldsymbol{K}_{\boldsymbol{ANG}\left( \boldsymbol{1-9} \right)}$ | Production of ANG(1-7) from ANG(1-9) | 5.22[1/h] | From model validation – Mok W. et al. [12] |
| $\boldsymbol{K}_{\boldsymbol{MAs}}^{\boldsymbol{on}}$ | Rate of ANG(1-7) binding to MAs receptors | 25 [ml/h/nmol] | From model validation – Mok W. et al. [12] |
| $\boldsymbol{K}_{\boldsymbol{MAs}}^{\boldsymbol{off}}$ | Rate of ANG(1-7) detachment from MAS receptors | 5.22[1/h] | From model validation – Mok W. et al. [12] |
| $\boldsymbol{K}_{\boldsymbol{AT}\boldsymbol{4}}^{\boldsymbol{on}}$ | Rate of ANGIV binding to AT4R receptors | 25 [ml/h/nmol] | From model validation – Mok W. et al. [12] |
| $\boldsymbol{K}_{\boldsymbol{AT}\boldsymbol{4}}^{\boldsymbol{off}}$ | Rate of ANGIV detachment from AT4R receptors | 5.22[1/h] | From model validation – Mok W. et al. [12] |
| $\boldsymbol{K}_{\boldsymbol{IL}\boldsymbol{6}}$ | Production rate of IL6 by activated macrophages | 0.5 [ml/h] | From model validation |
| $\boldsymbol{\gamma}_{\boldsymbol{IL}\boldsymbol{6}}$ | Degradation rate of IL6 | 6.3 x10^-4^  [1/h] | From model validation |
| $\boldsymbol{K}_{\boldsymbol{IL}\boldsymbol{6}}^{\boldsymbol{on}}$ | Rate of IL6 binding to IL6 receptors | 15 [ml/h/nmol] | From model validation – Mok W. et al. [12] |
| $\boldsymbol{K}_{\boldsymbol{IL}\boldsymbol{6}}^{\boldsymbol{off}}$ | Rate of IL6 detachment from IL6 receptors | 5.22[1/h] | From model validation – Mok W. et al. [12] |
| $\boldsymbol{h}_{\boldsymbol{IL}\boldsymbol{6}\boldsymbol{R}}$ | The half-life for IL6 receptor | 1.5 [min] | From model validation – same as $h_{AT1R-ANGII}$ |
| $\boldsymbol{S}_{\boldsymbol{sIL}\boldsymbol{6}\boldsymbol{R}}$ | Source production of sIL6R | 0.57[fmol/ml/h] | From model validation – same as $s_{Renin}$ |
| $\boldsymbol{K}_{\boldsymbol{sIL}\boldsymbol{6}\boldsymbol{R}}^{\boldsymbol{on}}$ | Rate of IL6 binding to soluble IL6 receptors | 17 [ml/h/nmol] | From model validation – Mok W. et al. [12] |
| $\boldsymbol{S}_{\boldsymbol{VEGF}}$ | Source production of VEGF | 0.235[fmol/ml/h] | From model validation – same as $\frac{s_{Renin}}{2}$ |
| $\boldsymbol{K}_{\boldsymbol{VEGF}}$ | Production rate of VEGF by IL6 bound on the soluble IL6 receptor | 5.22[1/h] | From model validation – Mok W. et al. [12] |
| $\boldsymbol{K}_{\boldsymbol{AT}\boldsymbol{1}\boldsymbol{R-MAsR}}$ | Degradation rate of pro-inflammatory cytokines by Mas receptor | 5.22[1/h] | From model validation – Mok W. et al. [17] |
| $\boldsymbol{K}_{\boldsymbol{AT}\boldsymbol{1}\boldsymbol{R-AT}\boldsymbol{2}\boldsymbol{R}}$ | Degradation rate of pro-inflammatory cytokines by AT2 receptor | 5.22[1/h] | From model validation – Mok W. et al. [12] |
| $\boldsymbol{S}_{\boldsymbol{AT}\boldsymbol{1}\boldsymbol{R}}$ | Source term for AT1R | 0.57[fmol/ml/h] | From model validation – same as $s_{Renin}$ |
| $\boldsymbol{S}_{\boldsymbol{AT}\boldsymbol{2}\boldsymbol{R}}$ | Source production of AT2R | 0.57[fmol/ml/h] | From model validation – same as $s_{Renin}$ |
| $\boldsymbol{S}_{\boldsymbol{MAsR}}$ | Source production of MAsR | 0.57[fmol/ml/h] | From model validation – same as $s_{Renin}$ |
| $\boldsymbol{S}_{\boldsymbol{AT}\boldsymbol{4}\boldsymbol{R}}$ | Source production of AT4R | 0.57[fmol/ml/h] | From model validation – same as $s_{Renin}$ |
| $\boldsymbol{S}_{\boldsymbol{IL}\boldsymbol{6}\boldsymbol{R}}$ | Source production of IL6R receptor | 0.57[fmol/ml/h] | From model validation – same as $s_{Renin}$ |
| $\boldsymbol{K}_{\boldsymbol{sIL}\boldsymbol{6}\boldsymbol{R}}$ | Conversion rate of IL6 receptor to soluble IL6 receptor | 5.22[1/h] | From model validation – Mok W. et al. [12] |
| $\boldsymbol{K}_{\boldsymbol{sIL}\boldsymbol{6}\boldsymbol{R}}^{\boldsymbol{off}}$ | Rate of IL6 detachment from soluble IL6 receptors | 2.22[1/h] | From model validation – Mok W. et al. [12] |
| $\boldsymbol{S}_{\boldsymbol{sACE}\boldsymbol{2}}$ | Source production of sACE2 | 0.57[fmol/ml/h] | From model validation – Mok W. et al. [12] |
| $\boldsymbol{K}_{\boldsymbol{sACE}\boldsymbol{2}}$ | Rate of soluble ACE2 receptor binding to the virus | 17 [ml/h/nmol] | From model validation – Mok W. et al. [12] |
| $\boldsymbol{K}_{\boldsymbol{Adam}\boldsymbol{17}}$ | Production rate of soluble ACE2 receptor by ACE2 receptor interaction through Adam17 | 5.22[1/h] | From model validation – Mok W. et al. [12] |
| $\boldsymbol{K}_{\boldsymbol{ACE}\boldsymbol{2-Virus}}^{\boldsymbol{on}}$ | Rate of virus binding to ACE2 receptors | 17 [ml/h/nmol] | From model validation – Mok W. et al. [12] |
| $\boldsymbol{K}_{\boldsymbol{ACE}\boldsymbol{2-Virus}}^{\boldsymbol{off}}$ | Rate of virus detachment from ACE2 receptors | 5.22[1/h] | From model validation – Mok W. et al. [12] |
| $\boldsymbol{\chi}_{\boldsymbol{N-IL}\boldsymbol{6}}$ | Production of neutrophils by IL6-R activation | 5.26[1/fmol/h] | From model validation- Dunster J.L. et al. [14] |
| $\boldsymbol{\chi}_{\boldsymbol{Ma-IL}\boldsymbol{6}}$ | Production of macrophages by IL6-R activation | 5.26[1/fmol/h] | From model validation- Dunster J.L. et al. [14] |
| $\boldsymbol{K}_{\boldsymbol{ec}}$ | Proliferation of endothelial cells | 5.22x10^-7^ [1/h] | From model validation |
| $\boldsymbol{K}_{\boldsymbol{ec}}^{\boldsymbol{b}}$ | Endothelial cell infection rate | 70 [ml/h/nmol] | From model validation |
| $\boldsymbol{S}_{\boldsymbol{v}}^{\boldsymbol{0}}$ | Vascular density of the normal lung | 70 [1/cm] | Mpekris F. et al [18] |
| $\boldsymbol{K}_{\boldsymbol{ACE}\boldsymbol{2}}$ | Production of ANG(1-7) by ANGII bound to ACE2 | 65.22[1/h] | Pilvankar et al. [9] |
| $\boldsymbol{S}_{\boldsymbol{ACE}\boldsymbol{2}}$ | Production rate of ACE2 by healthy endothelial and epithelial cells | 0.57[fmol/ml/h] | From model validation – same as $s_{Renin}$ |
| $\boldsymbol{V}_{\boldsymbol{O}\boldsymbol{2}}^{\boldsymbol{max}}$ | oxygen uptake rate | 3700 [mlO_2_/min] | Weibel E.R. et al [19] |
| $\boldsymbol{P}_{\boldsymbol{A}}$ | Partial pressure of oxygen in alveolar air | 100 [mmHg] | T.K. Roy, T.W. Secomb [20] |
| $\boldsymbol{K}_{\boldsymbol{02}}^{\boldsymbol{0}}$ | Krogh permeability coefficient KO2 | 3.3x10^-8^ [cm^2^/min/mmHg] | Weibel E.R. et al [19] |
| $\boldsymbol{S(A)}$ | Alveolar gas exchange areas | 130 [m^2^] | Weibel E.R. et al [19] |
| $\boldsymbol{S(c)}$ | Capillary gas exchange areas | 115 [m^2^] | Weibel E.R. et al [19] |
| $\boldsymbol{\tau}_{\boldsymbol{hb}}$ | Harmonic mean thickness of the air–blood barrier | 1 [μm] | Weibel E.R. et al [19] |
| $\boldsymbol{\theta}_{\boldsymbol{O}\boldsymbol{2}}$ | Oxygen unloading conductance of blood | 1.8[mlO_2_/ml/min/mmHg] | Roy T.K., Secomb T.W. [20] |
| $\boldsymbol{V(c)}$ | Lung blood volume | 194 [ml] | Roy T.K., Secomb T.W. [20] |
| $\boldsymbol{n}$ | Hill coefficient | 2.7 | Roy T.K., Secomb T.W. [20] |
| $\boldsymbol{P}_{\boldsymbol{50}}$ | Oxygen tension when the binding sites are 50 percent saturated. | 26.3 [mmHg] | Roy T.K., Secomb T.W. [20] |
| $\boldsymbol{K}_{\boldsymbol{in}}$ | Rate of release of replicated virus | 0.4 x10^-7^ [1/h] | Mahasa KJ et al.[16] |
| $\boldsymbol{d}_{\boldsymbol{AT}\boldsymbol{1}\boldsymbol{R}}$ | Degradation rate of AT1R | 6.3x10^-4^[1/h] | From model validation- Dunster J.L. et al. [14] |
| $\boldsymbol{d}_{\boldsymbol{AT}\boldsymbol{2}\boldsymbol{R}}$ | Degradation rate of AT2R | 6.3x10^-4^[1/h] | From model validation- Dunster J.L. et al. [14] |
| $\boldsymbol{d}_{\boldsymbol{MAsR}}$ | Degradation rate of MAsR | 6.3x10^-4^[1/h] | From model validation- Dunster J.L. et al. [14] |
| $\boldsymbol{d}_{\boldsymbol{AT}\boldsymbol{4}\boldsymbol{R}}$ | Degradation rate of AT4R | 6.3x10^-4^[1/h] | From model validation- Dunster J.L. et al. [14] |
| $\boldsymbol{d}_{\boldsymbol{IL}\boldsymbol{6}\boldsymbol{R}}$ | Degradation rate of IL6R | 6.3x10^-4^[1/h] | From model validation- Dunster J.L. et al. [14] |
| $\boldsymbol{d}_{\boldsymbol{sACE}\boldsymbol{2}}$ | Degradation rate of soluble ACE2 receptor | 6.3x10^-4^[1/h] | From model validation- Dunster J.L. et al. [14] |
| $\boldsymbol{K}_{\boldsymbol{IL}\boldsymbol{6-TN}}$ | Production rate of IL6 by Naïve T cells | 0.02554[mol/pg/s] | From model validation - Lai, X., & Friedman A. [21] |
| $\boldsymbol{K}_{\boldsymbol{IL}\boldsymbol{6-In}}$ | Production rate of IL6 by infected epithelial cells | 0.02554[mol/pg/s] | From model validation - Lai, X., & Friedman A. [21] |
| $\boldsymbol{K}_{\boldsymbol{IL}\boldsymbol{6-TE}}$ | Production rate of IL6 by Activated T cells | 0.02554[mol/pg/s] | From model validation - Lai, X., & Friedman A. [21] |
| $\boldsymbol{K}_{\boldsymbol{IL}\boldsymbol{6-iEC}}$ | Production rate of IL6 by infected endothelial cells | 0.02554[mol/pg/s] | From model validation - Lai, X., & Friedman A. [21] |
| $\boldsymbol{\gamma}_{\boldsymbol{VEGF}}$ | Production rate of VEGF by hypoxia | 0.0152[mol/ml/h] | From model validation |
| $\boldsymbol{K}_{\boldsymbol{AT}\boldsymbol{1}\boldsymbol{R}}$ | Production of pro-inflammatory cytokines by AT1R activation | 4.2x10^2^ [pg/h/fmol] | From model validation |
| $\boldsymbol{K}_{\boldsymbol{c-IL}\boldsymbol{6}}$ | Production of pro-inflammatory cytokines by IL6-R activation | 0.03 [ml/h/fmol] | From model validation |
| $\boldsymbol{K}_{\boldsymbol{c-EC}}$ | Production of pro-inflammatory cytokines by healthy endothelial cells | 1 [pg] | From model validation |
| $\boldsymbol{K}_{\boldsymbol{c-H}}$ | Production of pro-inflammatory cytokines by healthy epithelial cells | 1 [pg] | From model validation |
| $\boldsymbol{K}_{\boldsymbol{HIn}}\boldsymbol{=}\boldsymbol{K}_{\boldsymbol{HIEC}}$ | proportion coefficients for production of infected epithelial and endothelial cells by neutrophils and pro/anti-inflammatory cytokines | 0.02554 [ml/pg] | From model validation |
| $\boldsymbol{h}_{\boldsymbol{T}}\boldsymbol{=}\boldsymbol{h}_{\boldsymbol{TE}}$=$\boldsymbol{h}_{\boldsymbol{ThE}}\mathbf{=}\boldsymbol{h}_{\boldsymbol{BE}}$ | Conversion rate of Naïve T and B cells to activated cells | 0.00254 [1/h] | From model validation - Lai, X., & Friedman A. [21] |
| $\boldsymbol{As}$ | Antigen strength | 1 | From model validation - Lai, X., & Friedman A. [21] |
| $\boldsymbol{K}_{\boldsymbol{T}}$ | Constant for blocking PD-1 inhibition | 1.365x10^-18^ [g/cm^3^] | From model validation - Lai, X., & Friedman A. [21] |
| $\boldsymbol{\varepsilon}$ | Degradation of activated T cells by PD1 bound to PDL1 | 0.01575 [cm^3^/g/s] | From model validation - Lai, X., & Friedman A. [21] |
| $\boldsymbol{d}_{\boldsymbol{H}}$ | Degradation rate PDL1 by healthy epithelial cells | 0.00215 [1/s] | From model validation - Lai, X., & Friedman A. [21] |
| $\boldsymbol{d}_{\boldsymbol{EC}}$ | Degradation rate PDL1 by endothelial healthy cells | 0.00215 [1/s] | From model validation - Lai, X., & Friedman A. [21] |
| $\boldsymbol{d}_{\boldsymbol{In}}$ | Degradation rate PDL1 by epithelial infected Cells | 0.00215 [1/s] | From model validation - Lai, X., & Friedman A. [21] |
| $\boldsymbol{d}_{\boldsymbol{TE}}$ | Degradation rate PD1 by Activated T cells | 0.00215x10^-3^ [1/s] | From model validation - Lai, X., & Friedman A. [21] |
| $\boldsymbol{d}_{\boldsymbol{TN}}$ | Degradation rate PD1 by Naïve T cells | 0.00215 [1/s] | From model validation - Lai, X., & Friedman A. [21] |
| $\boldsymbol{d}_{\boldsymbol{N}}$ | Degradation rate PD1 by neutrophils | 0.00215 [1/s] | From model validation - Lai, X., & Friedman A. [21] |
| $\boldsymbol{d}_{\boldsymbol{Ma}}$ | Degradation rate PD1 by macrophages | 0.00215 [1/s] | From model validation - Lai, X., & Friedman A. [21] |
| a_PL_ | Association of PD-1 with PD-L1 | 0.258[mm^3/g/s] | From model validation - Lai, X., & Friedman A. [21] |
| $\boldsymbol{d}_{\boldsymbol{Q}}$ | Dissociation rate of PD-L1 from PD-1 | 0.1 [1/d] | From model validation - Lai, X., & Friedman A. [21] |
| γ_A_ | Source of anti-PD1 | 1x10^-10^[g/cm^3/d] | From model validation - Lai, X., & Friedman A. [21] |
| µ_PD1_ | Efficiency of PD1 blocking by ICI | 0.00215x10^-3^[1/s] | From model validation - Lai, X., & Friedman A. [21] |
| $\boldsymbol{d}_{\boldsymbol{A}}$ | Degradation rate of anti-PD1 | 0.0462 [1/d] | From model validation - Lai, X., & Friedman A. [21] |
| $\boldsymbol{\lambda}_{\boldsymbol{H}}$ | Production rate of PDL1 by healthy epithelial cells | 0.154 [1/s] | From model validation - Lai, X., & Friedman A. [21] |
| $\boldsymbol{\lambda}_{\boldsymbol{EC}}$ | Production rate of PDL1 by healthy endothelial cells | 0.154 [1/s] | From model validation - Lai, X., & Friedman A. [21] |
| $\boldsymbol{\lambda}_{\boldsymbol{In}}$ | Production rate of PDL1 by infected endothelial cells | 0.154 [1/s] | From model validation - Lai, X., & Friedman A. [21] |
| $\boldsymbol{\lambda}_{\boldsymbol{TE}}$ | Production rate of PD1 by activated T cells | 0.154 [1/s] | From model validation - Lai, X., & Friedman A. [21] |
| $\boldsymbol{\lambda}_{\boldsymbol{TN}}$ | Production rate of PD1 by Naïve T cells | 0.154 [1/s] | From model validation - Lai, X., & Friedman A. [21] |
| $\boldsymbol{\lambda}_{\boldsymbol{N}}$ | Production rate of PD1 by neutrophils | 0.154 [1/s] | From model validation - Lai, X., & Friedman A. [21] |
| $\boldsymbol{\lambda}_{\boldsymbol{Ma}}$ | Production rate of PD1 by macrophages | 0.154 [1/s] | From model validation - Lai, X., & Friedman A. [21] |
| $\boldsymbol{h}_{\boldsymbol{PDL}\boldsymbol{1}}$ | Production rate of the PD1 ligand by Effector (Activated) T | 0.154 [1/s] | From model validation - Lai, X., & Friedman A. [21] |
| $\boldsymbol{\mu}_{\boldsymbol{PD}\boldsymbol{1}}$ | Degradation rate of PD1 by anti-PD1 | 0.00215 x10^-3^ [1/s] | From model validation - Lai, X., & Friedman A. [21] |
| $\boldsymbol{a}_{\boldsymbol{PL}}$ | rate of PD1 binding to PDL1 | 0.258 [mm^3^/g/s] | From model validation - Lai, X., & Friedman A. [21] |
| $\boldsymbol{\gamma}_{\boldsymbol{A}}$ | Source term of anti-PD1 | 1x10^-10^ [g/cm^3^/d] | From model validation - Lai, X., & Friedman A. [21] |
| $\boldsymbol{\mu}_{\boldsymbol{A}}$ | Degradation rate of anti-PD1 by PD1 | 6.87x10^6^ [cm^3^/g/d] | From model validation - Lai, X., & Friedman A. [21] |
| $Q_{liver}$ | Blood flow rate of liver (hepatic portal vein from G.I. and spleen, and hepatic artery) | 800 [ml/min] | [22] |
| $Q_{spleen}$ | Blood flow rate of spleen | 138 [ml/min] | [22] |
| $Q_{G.I.}$ | Blood flow rate of G.I. | 468 [ml/min] | [22] |
| $Q_{kidney}$ | Blood flow rate of kidney | 630 [ml/min] | [22] |
| $Q_{Torso}$ | Blood flow rate of Torso | 220 [ml/min] | From model validation - [22] |
| $Q_{lower body}$ | Blood flow rate of lower body | 413 [ml/min] | From model validation - [22] |
| $Q_{uppe body}$ | Blood flow rate of upper body | 138 [ml/min] | From model validation - [22] |
| $Q_{brain}$ | Blood flow rate of brain | 300 [ml/min] | From model validation - [22] |
| $Q_{cardic vessels}$ | Blood flow rate of cardiac vessels | 120 [ml/min] | From model validation - [22] |
| $L_{liver}$ | Lymphatic flow rate of liver | 8.7x10^-2^ [ml/min] | [22] |
| $L_{spleen}$ | Lymphatic flow rate of spleen | 8.7x10^-4^ [ml/min] | [22] |
| $L_{G.I.}$ | Lymphatic flow rate of G.I. | 3.0 x 10^-1^ [ml/min] | [22] |
| $L_{kidney}$ | Lymphatic flow rate of kidney | 1x 10^-3^ [ml/min] | From model validation - [22] |
| $L_{Torso}$ | Lymphatic flow rate of torso | 4.3x 10^-3^ [ml/min] | From model validation - [22] |
| $L_{lower body}$ | Lymphatic flow rate of lower body | 1x 10^-3^ [ml/min] | From model validation - [22] |
| $L_{uppe body}$ | Lymphatic flow rate of upper body | 2.6 x 10^-2^ [ml/min] | From model validation - [22] |
| $L_{brain}$ | Lymphatic flow rate of brain | 1x 10^-3^ [ml/min] | From model validation - [22] |
| $L_{cardic vessels}$ | Lymphatic flow rate of cardiac vessels | 4.3x 10^-3^ [ml/min] | From model validation - [22] |
| $L_{Hlung}$ | Lymphatic flow rate of normal part of lung | 4.3x 10^-2^ [ml/min] | [22] |
| $L_{Tlung}$ | Lymphatic flow rate of tumor part of lung | 2x 10^-2^ [ml/min] | From model validation - [22] |
| $V_{liver}$ | Averaged vascular volume of liver | 180.9 [ml] | [22] |
| $V_{spleen}$ | Averaged vascular volume of spleen | 17 [ml] | [22] |
| $V_{G.I.}$ | Averaged vascular volume of GI system | 43 [ml] | [22] |
| $V_{kidney}$ | Averaged vascular volume of kidney | 28.4 [ml] | [22] |
| $V_{Torso}$ | Averaged vascular volume of torso | 462 [ml] | From model validation |
| $V_{lower body}$ | Averaged vascular volume of lower body | 700 [ml] | From model validation |
| $V_{uppe body}$ | Averaged vascular volume of upper body | 150 [ml] | From model validation |
| $V_{brain}$ | Averaged vascular volume of brain | 150 [ml] | From model validation |
| $V_{cardic vessels}$ | Averaged vascular volume of cardiac vessels | 100 [ml] | From model validation |
| $V_{Hlung}$ | Averaged vascular volume of normal part of lung | 99.9 [ml] | [22] |
| $V_{Tlung}$ | Averaged vascular volume of normal part of lung | 50 [ml] | From model validation |
| $a_{i}$ | Attachment rate of virus to ACE2 | 1x 10^-3^ [1/(mM.min)] | From model validation |
| $a'$ | Attachment rate of virus to sACE2 | 8.5 [ml/h/nmol] | From model validation |
| $d_{i}$ | Detachment rate of virus from endothelium | 6.7x 10^-4^ [1/min] | From model validation |
| $mt_{i}$ | Maximum production of micro-thrombus (ACE2) | 7x 10^-3^ [mM/min] | From model validation |
| $mt'_{i}$ | Maximum production of micro-thrombus (IL6) | 7x 10^-3^ [mM/min] | From model validation |
| *mt_i_’’* | Maximum production of micro-thrombus (cytokines) | 7x 10^-3^ [mM/min] | From model validation |
| $mt'''$ | Maximum production of micro-thrombus (NET) | 7x 10^-3^ [mM/min] | From model validation |
| $\varepsilon_{i}$ | Dissolution of micro-thrombi due to thrombolysis | 7x 10^-5^ [1/min] | From model validation |
| $dm_{liver}$ | Attachment rate of micro-thrombus to vessels in liver | 6.9x 10^-6^ [1/min] | From model validation |
| $dm_{i}$ | Attachment rate of micro-thrombus to vessels in other organs | 6.9x 10^-6^ [1/min] | From model validation |
| $p_{\text{lung}}$ | Proliferation rate of internalized virus in lung | 7x 10^-3^ [1/min] | From model validation |
| $p_{\text{liver}}$ | Proliferation rate of internalized virus in liver | 7x 10^-3^ [1/min] | From model validation |
| $p_{\text{spleen}}$ | Proliferation rate of internalized virus in spleen | 0.7x 10^-3^ [1/min] | From model validation |
| $p_{\text{upper} \text{body}}$ | Proliferation rate of internalized virus in upper body | 7.6x 10^-3^ [1/min] | From model validation |
| $p_{\text{Torso}}$ | Proliferation rate of internalized virus in torso | 14x 10^-3^ [1/min] | From model validation |
| $p_{\text{lower body}}$ | Proliferation rate of internalized virus in lower body | 6.3x 10^-3^ [1/min] | From model validation |
| $p_{\text{intestine}}$ | Proliferation rate of internalized virus in intestine | 4.9x 10^-3^ [1/min] | From model validation |
| $p_{\text{brain}}$ | Proliferation rate of internalized virus in brain | 9.7x 10^-3^ [1/min] | From model validation |
| $p_{\text{kidney}}$ | Proliferation rate of internalized virus in kidney | 7.6x 10^-3^ [1/min] | From model validation |
| $p_{\text{cardiac vessels}}$ | Proliferation rate of internalized virus in cardiac vessels | 12.5x 10^-3^ [1/min] | From model validation |
| $k_{acg}$ | Micro-thrombus-inhibiting coefficient of anti-coagulation drugs for virus-ACE-2-induced coagulation | 0.5 | From model validation |
| $k'_{acg}$ | Micro-thrombus-inhibiting coefficient of anti-coagulation drugs for IL6-induced coagulation | 0.5 | From model validation |
| $k''_{acg}$ | Micro-thrombus-inhibiting coefficient of anti-coagulation drugs for cytokine storm-induced coagulation | 0.5 | From model validation |
| $k'''_{acg}$ | Micro-thrombus-inhibiting coefficient of anti-coagulation drugs for NET-induced coagulation | 0.5 | From model validation |
| $k_{v}$ | Degradation rate of free virus by Antibody | 4x10^-6^ [1/d/nmol] | From model validation – Lee et al [23] |
| $k_{A}$ | Clearance rate of antibody by free viruses | 0.04 [1/d/nmol] | Lee et al [23] |
| $k_{T,IEC}$ | Production rate of the micro-thrombus by infected endothelial cells | 1.4x 10^-3^ [mM/min] | From model validation |
|  | Production rate of the micro-thrombus by infected endothelial cells and effector CD8+ T cells | 1.4x 10^-3^ [mM/min] | From model validation |
| $k_{A,v}$ | Clearance rate of antibody by free viruses | 1x 10^-3^ [1/(mM.min)] | From model validation |
| $k_{A,IEC}$ | Degradation rate of antibody by infected endothelial cells | 2.1x10^-10^ [ml/h/nmol] | From model validation |
| $k_{a}$ | Natural decay rate of antibody | 4x10^-7^ [1/d ] | From model validation |
| $k_{c,innate}$ | Production rate of the cytokine by innate immune cells | 2.3x10^-9^ [ml/h/nmol] | From model validation |
| $k_{c,IEC}$ | Production rate of the cytokine by infected endothelial cells | 2.3x10^-9^ [ml/h/nmol] | From model validation |
| $K_{c-IL6}$ | Production rate of the cytokine by IL6 bound to sIL6R | 0.03 [ml/h/fmol] | From model validation |
| $d_{s}$ | Degradation rate of pro-inflammatory cytokines | 8.3x10^-1^ [1/h] | Smith A.M. et al [13] |
| $K_{F}$ | Killing rate of infected cells by NK cells | 4.2 [1/d] | A. Haghnegahdar et all [24] |
|  | proportion coefficient for production of infected endothelial cells by neutrophils and pro/anti-inflammatory cytokines | 2.1x10^-2^ [pg/h] | Dunster J.L. et al. [14] |
| $K_{InM} = K_{IECM}$ | Killing rate of infected cells by Memory cells | 4.2 [1/d] | From model validation |
|  | Degradation rate of antibody by infected endothelial and epithelial cells | 2.1x10^-10^ [ml/h] | From model validation |
|  | Production rate of the micro-thrombus by infected endothelial and epithelial cells | 1.4x 10^-3^ [mM/min] | From model validation |
|  | Production rate of the micro-thrombus by infected endothelial and epithelial cells and effector CD8+ T cells | 1.4x 10^-3^ [mM/min] | From model validation |
| $S_{DC}$ | source term of dendritic cells | 1 [1/d] | Lee et al [23] |
| $\beta_{DC}$ | Conversion rate of immature dendritic cells to APC | 1x10^-2^ [1/d/M] | Lee et al [23] |
| $d_{DC}$ | Death rate of dendritic cells | 1x10^-3^ [1/d] | Lee et al [23] |
| $S_{ThN}$ | source term of Naïve CD4+ T cells | 4x10^-4^ [1/d] | Lee et al [23] |
| $d_{ThN}$ | Death rate of Naïve CD4+ T cells | 0.75 [1/d] | Lee et al [23] |
| $\boldsymbol{\pi}_{\boldsymbol{ThM}}$ | conversion rate of effector CD4+ T cells to memory CD4+ T cells | - | From model validation |
| $S_{TN}$ | source term of Naïve CD8+ T cells | 750 [1/d] | Lee et al [23] |
| $d_{TN}$ | Death rate of Naïve CD8+ T cells | 0.75 [1/d] | Lee et al [23] |
| $\boldsymbol{\pi}_{\boldsymbol{TM}}$ | conversion rate of effector CD8+ T cells to memory CD8+ T cells | - | From model validation |
| $S_{B}$ | source term of Naïve B cells | 2 [1/d] | Lee et al [23] |
| $d_{B}$ | Death rate of Naïve B cells | 0.002 [1/d] | Lee et al [23] |
| $\boldsymbol{\pi}_{\boldsymbol{s}}$ | differentiation rate of activated B cells into short-lived antibody-secreting plasma cells. | 1e-3 [1/d] | Lee et al [23] |
| $\boldsymbol{\pi}_{\boldsymbol{L}}$ | differentiation rate of activated B cells into long-lived antibody-secreting plasma cells. | 8e-9 [1/d] | Lee et al [23] |
| $\boldsymbol{\rho}_{\boldsymbol{B}}$ | the proliferation rate of activated B cells | 2.6 [1/d] | Lee et al [23] |
| $\boldsymbol{\delta}_{\boldsymbol{L}}$ | death rate of long-lived antibody-secreting plasma cell | 3e-2 [1/d] | Lee et al [23] |
| $\boldsymbol{\delta}_{\boldsymbol{s}}$ | death rate of short-lived antibody-secreting plasma cell | 0.1 [1/d] | Lee et al [23] |
| $\boldsymbol{\delta}_{\boldsymbol{ThM}}$ | death rate of memory CD4+ cells | 3e-4 [1/d] | From model validation |
| $\boldsymbol{\delta}_{\boldsymbol{TM}}$ | death rate of memory CD8+ cells | 3e-4 [1/d] | From model validation |
| $\boldsymbol{S}^{\boldsymbol{Va}}$ | Source term for Free vaccine | 1[M/d] | From model validation |
| $\boldsymbol{K}_{\boldsymbol{cell}\mathbf{-}\boldsymbol{Vaccine}}^{\boldsymbol{on}}$ | Binding of the vaccine particles to Healthy cells | 17 [ml/h] | From model validation |
| $\boldsymbol{K}_{\boldsymbol{cell}\mathbf{-}\boldsymbol{Vaccine}}^{\boldsymbol{off}}$ | unbinding of the vaccine particles to Healthy cells | 5.22[m^3^/h] | From model validation |
| $\boldsymbol{K}_{\boldsymbol{dVa}}$ | Degradation rate of vaccine particles | 8.1e-4 [1/d] | From model validation |
| $\boldsymbol{K}_{\boldsymbol{intVa}}$ | internalization of the bound vaccine particles into the cells | 5.78e-2[1/s] | From model validation |
| $\boldsymbol{K}_{\boldsymbol{dVab}}$ | degradation of the bound vaccine particles | 4e-4 [1/d] | From model validation |
| $\boldsymbol{K}_{\boldsymbol{Tran}}$ | DNA transcription to mRNA of the internalize vaccine particles | 6.21 e-4 [1/s] | From model validation |
| $\boldsymbol{K}_{\boldsymbol{protein}}$ | production of viral protein or viral antigen from the translation of the mRNA | 8.52 e-5 [1/s] | From model validation |
| $\boldsymbol{d}_{\boldsymbol{Vaint}}$ | constant degradation of internalized vaccine | 3.4 e-4 [mole/m^3^/s] | From model validation |
| $\boldsymbol{d}_{\boldsymbol{VaTran}}$ | constant degradation of translation DNA | 1.5 e-4 [mole/m^3^/s] | From model validation |
| $\boldsymbol{d}_{\boldsymbol{Vprotein}}$ | constant degradation of viral proteins | 6.5 e-5 [mole/m^3^/s] | From model validation |
| $\boldsymbol{K}_{\boldsymbol{cell}\mathbf{-}\boldsymbol{Vaccine}}^{\boldsymbol{on}}$ | Binding of the vaccine particles to healthy cells | Baseline:17[ml/h] |  |
| $\boldsymbol{K}_{\boldsymbol{cell}\mathbf{-}\boldsymbol{Vaccine}}^{\boldsymbol{off}}$ | unbinding of the vaccine particles to healthy cells | 6.22[m^3^/h] |  |
| $\boldsymbol{K}_{\boldsymbol{dVa}}$ | Degradation rate of vaccine particles | 9.4e-4 [1/d] |  |
| $\boldsymbol{K}_{\boldsymbol{intVa}}$ | internalization of the bound vaccine particles into the cells | 4.78e-2[1/s] |  |
| $\boldsymbol{K}_{\boldsymbol{dVab}}$ | degradation of the bound vaccine particles | 4.5e-4 [1/d] |  |
| $\boldsymbol{K}_{\boldsymbol{protein}}$ | production of viral protein or viral antigen from the translation of the mRNA | 8.0e-5 [1/s] |  |
| $\boldsymbol{d}_{\boldsymbol{Vaint}}$ | constant degradation of internalized vaccine | 3.8 e-4 [mole/m^3^/s] |  |
| $\boldsymbol{d}_{\boldsymbol{VaTran}}$ | constant degradation of translation DNA | 2 e-4 [mole/m^3^/s] |  |
| $\boldsymbol{d}_{\boldsymbol{Vprotein}}$ | constant degradation of viral proteins | 7e-5 [mole/m^3^/s] |  |

**Supplementary Table 2.** Values of model parameters related to treatment.

| **Parameter** | **Description** | **non-treatment** | **Remdesivir** | **Molnupiravir** | **NMV/r** |
| --- | --- | --- | --- | --- | --- |
| $\boldsymbol{K}_{\boldsymbol{in}}$ | Rate of release of replicated virus | 2.30E-07 [1/h] | 3.20E-09[1/h] | 2.10E-09[1/h] | 4.20E-09[1/h] |

**Supplementary Table 3.** Values of model parameters related to perturbations to the immune response.

|  | Parameter | Description | Value |
| --- | --- | --- | --- |
| Immune System Response | $S_{ThN}$ | source term of Naïve CD4+ T cells | 4x10^-4^ [1/d] |
|  | $d_{ThN}$ | Death rate of Naïve CD4+ T cells | 0.75 [1/d] |
|  | $\pi_{ThM}$ | conversion rate of effector CD4+ T cells to memory CD4+ T cells | 1x10-2 [1/d/M] |
|  | $S_{TN}$ | source term of Naïve CD8+ T cells | 750 [1/d] |
|  | $d_{TN}$ | Death rate of Naïve CD8+ T cells | 0.75 [1/d] |
|  | $\pi_{TM}$ | conversion rate of effector CD8+ T cells to memory CD8+ T cells | 1x10-2 [1/d/M] |
| Production rate of pro-inflammatory cytokines | $K_{c-EC}$ | Production of pro-inflammatory cytokines by healthy endothelial cells | 1 [pg] |
|  | $K_{c-H}$ | Production of pro-inflammatory cytokines by healthy epithelial cells | 1 [pg] |
| Production rates of microthrombus | $mt_{i}$ | Maximum production of micro-thrombus (ACE2) | 7x 10^-3^ [mM/min] |
|  | $mt'_{i}$ | Maximum production of micro-thrombus (IL6) | 7x 10^-3^ [mM/min] |
|  | *mt_i_’’* | Maximum production of micro-thrombus (cytokines) | 7x 10^-3^ [mM/min] |

**Supplementary Table 4.** Values of model parameters related to perturbations to the immune response.

| Parameter | Description | Older | Younger |
| --- | --- | --- | --- |
| $\boldsymbol{\tau}_{\boldsymbol{hb}}$ | Harmonic mean thickness of the air–blood barrier | 100 [μm] | 1 [μm] |
| $\boldsymbol{K}_{\boldsymbol{c-EC}}$ | Production rate of pro-inflammatory cytokines by healthy endothelial cells | 100 [pg] | 1 [pg] |
| $\boldsymbol{K}_{\boldsymbol{c-H}}$ | Production rate of pro-inflammatory cytokines by healthy epithelial cells | 100 [pg] | 1 [pg] |
| $\boldsymbol{S}_{\boldsymbol{TN}}$ | Source production of naïve T cells | 0.00235 [pg/ml/h] | 0.235 [pg/ml/h] |
| $\boldsymbol{h}_{\boldsymbol{TE}}$ | Conversion rate of Naïve T cells to activated T cells | 0.0000254 [1/h] | 0.00254 [1/h] |
| $mt_{i}$ | Maximum production rates of micro-thrombus (ACE2) | 7x 10^-1^ [mM/min] | 7x 10^-3^ [mM/min] |
| $mt'_{i}$ | Maximum production rates of micro-thrombus (IL6) | 7x 10^-1^ [mM/min] | 7x 10^-3^ [mM/min] |
| *mt_i_’’* | Maximum production rates of micro-thrombus (cytokines) | 7x 10^-1^ [mM/min] | 7x 10^-3^ [mM/min] |

**Supplementary Table 5.** Categorization of Model Findings in COVID-19 Research. The table delineates the findings derived from our mathematical model into three categories: (1) Model predictions consistent with existing data, (2) Novel model predictions requiring clinical validation, and (3) Model predictions potentially contradicting existing data.

| Model predictions consistent with existing data | Novel model predictions requiring clinical validation | Model predictions potentially contradicting existing data |
| --- | --- | --- |
| Viral neutralization by vaccine induced antibodies in setting of infection with omicron variants from BA.1 to BA 4/5 [2-5] | Our results confirm that antiviral therapy confers benefit even in the setting of vaccination and that effective antiviral therapy is more important to outcome in the setting of variants which evade vaccine induced immunity. [25] | We find that when pro-inflammatory cytokine production is increased, the decreased viral load comes at the cost of poorer gas exchange. Increasing the effectiveness of anti-viral therapy in the setting of increased inflammatory cytokine production attenuates the adverse effects on gas exchange (consistent with the more rapid decrease in inflammatory stimulus). [26, 27] |
| The proportion of patients that recovered from COVID-19 having been treated with antivirals, remdesivir, molnupiravir and nirmatrelvir+ritonavir (NMV/r) [6-8] | Effective antiviral therapy is more important to treatment outcome in the setting of variants which evade vaccine induced immunity [28, 29]. | When neutrophil population is set to zero, the model predicts worsening oxygen saturation with elevated levels of cytokine production, as in the case with neutrophils present [30-32] |
| Viral load rebound after antiviral therapy [33] | High rates of immune cell activation and inflammatory cytokine production result in lower viral load – and that viral control is augmented by effective antiviral therapy [34-36] | Very high initial viral loads can result in high peak viral loads even at very high levels of expression of proinflammatory cytokines  [37-39] |

**Equations of the Mathematical Modeling Framework**

1. Pharmacokinetic-pharmacodynamic (PK/PD) model

The PK/PD model has been formulated to incorporate major organs: lung, heart, liver, brain, spleen, gastro-intestinal, upper body, lower body, torso, cardiac vessels and the tumor . The PK/PD model allows for transport of viral particles, antibodies, cytokines and micro-thrombi among these compartments.

- 1. Heart compartment

In this model, heart circulates blood through all the compartments. The heart compartment includes arterial and venous blood flows for free viruses, micro-thrombosis, cytokines and antibody. aHeart and vHeart refer to the left and right heart ventricles, respectively.

- - 1. Free virus recirculation in arterial and venous blood flows into left and right heart ventricles, $v_{aHeart}$and $v_{vHeart}$,

| $\frac{dv_{aHeart}}{dt}= \left(\begin{matrix} ( Q_{Hlung}- L_{Hlung}) v_{Hlung}+( Q_{Tlung}- L_{Tlung}) v_{Tlung} \\ -(Q_{liver}+L_{spleen}+L_{G.I.}+Q_{kidney} \\ +Q_{Torso}+Q_{lower body}+Q_{uppe body}+Q_{brain} \\ +Q_{cardic vessels}) v_{aHeart}-k_{v} v_{aHeart} A_{aHeart} V_{aHeart} \end{matrix}\right)/V_{aHeart}$ | (1a) |
| --- | --- |

where the first group of terms represents the recirculation in arterial blood flow free viruses and the last term describes the effect of the antibodies on the virus reproduction

| $\frac{dv_{vHeart}}{dt}= \left(\begin{matrix} ( Q_{liver}- L_{liver}) v_{liver}+( Q_{Torso}- L_{Torso}) v_{Torso} \\ +( Q_{upper body}- L_{upper body}) v_{upper body} \\ +( Q_{lower body}- L_{lower body}) v_{lower body} \\ +( Q_{cardic vessels}- L_{cardic vessels}) v_{cardic vessels} \\ +( Q_{brain}- L_{brain}) v_{brain} \\ +( Q_{kidney}- L_{kidney}) v_{kidney} \\ - ( Q_{Hlung}+Q_{Tlung})v_{vHeart}-k_{v} v_{vHeart} A_{vHeart} V_{vHeart} \end{matrix}\right)/V_{vHeart}$ | (1b) |
| --- | --- |

where the first group of terms represents the recirculation in venous blood flow for free viruses and the last term describes the effect of the antibodies on the virus reproduction

- - 1. Arterial and venous recirculation of micro-thrombi, $T_{aHeart}$ and $T_{vHeart}$,

| $\frac{dT_{aHeart}}{dt}= \left(\begin{matrix} ( Q_{Hlung}- L_{Hlung}) T_{Hlung}+( Q_{Tlung}- L_{Tlung}) T_{Tlung} \\ -(Q_{liver}+L_{spleen}+L_{G.I.}+Q_{kidney} \\ +Q_{Torso}+Q_{lower body}+Q_{uppe body}+Q_{brain} \\ +Q_{cardic vessels}) T_{aHeart} \end{matrix}\right)/V_{aHeart}$ | (2a) |
| --- | --- |

where the terms represent the recirculation in arterial blood flow for micro-thrombosis

| $\frac{dT_{vHeart}}{dt}= \left(\begin{matrix} ( Q_{liver}- L_{liver}) T_{liver}+( Q_{Torso}- L_{Torso}) T_{Torso} \\ +( Q_{upper body}- L_{upper body}) T_{upper body} \\ +( Q_{lower body}- L_{lower body}) T_{lower body} \\ +( Q_{cardic vessels}- L_{cardic vessels}) T_{cardic vessels} \\ +( Q_{brain}- L_{brain}) T_{brain} \\ +( Q_{kidney}- L_{kidney}) T_{kidney} \\ - ( Q_{Hlung}+Q_{Tlung})T_{vHeart} \end{matrix}\right)/V_{vHeart}$ | (2b) |
| --- | --- |
| where the terms represent the recirculation in venous blood flow for micro-thrombosis |  |

- - 1. Arterial and venous recirculation of antibody, $A_{aHeart}$ and $A_{vHeart}$,

| $\frac{dA_{aHeart}}{dt}= \left(\begin{matrix} ( Q_{Hlung}- L_{Hlung}) A_{Hlung}+ ( Q_{Tlung}- L_{Tlung}) A_{Tlung} \\ -(Q_{liver}+L_{spleen}+L_{G.I.}+Q_{kidney} \\ +Q_{Torso}+Q_{lower body}+Q_{uppe body}+Q_{brain} \\ +Q_{cardic vessels}) A_{aHeart} \\ - k_{A,v} v_{aHeart} A_{aHeart} V_{aHeart} \end{matrix}\right)/V_{aHeart}$ | (3a) |
| --- | --- |

where the first group of terms represents the recirculation in arterial blood flow for antibody and the last term describes the effect of the antibodies on the virus reproduction

| $\frac{dA_{vHeart}}{dt}= \left(\begin{matrix} ( Q_{liver}- L_{liver}) A_{liver}+( Q_{Torso}- L_{Torso}) A_{Torso} \\ +( Q_{upper body}- L_{upper body}) A_{upper body} \\ +( Q_{lower body}- L_{lower body}) A_{lower body} \\ +( Q_{cardic vessels}- L_{cardic vessels}) A_{cardic vessels} \\ +( Q_{brain}- L_{brain}) A_{brain} \\ +( Q_{kidney}- L_{kidney}) A_{kidney} \\ - (Q_{Hlung}+Q_{Tlung})A_{vHeart} \\ - k_{A,v} v_{vHeart} A_{vHeart} V_{vHeart} \end{matrix}\right)/V_{vHeart}$ | (3b) |
| --- | --- |
| where the first group of terms represents the recirculation in venous blood flow for antibody and the last term describes the effect of the antibodies on the virus reproduction |  |

- - 1. Arterial and venous recirculation of cytokines, $c_{aHeart}$ and $c_{vHeart}$,

| $\frac{dc_{aHeart}}{dt}= \left(\begin{matrix} ( Q_{Hlung}- L_{Hlung}) c_{Hlung}+( Q_{Tlung}- L_{Tlung}) c_{Tlung} \\ -(Q_{liver}+L_{spleen}+L_{G.I.}+Q_{kidney} \\ +Q_{Torso}+Q_{lower body}+Q_{uppe body}+Q_{brain} \\ +Q_{cardic vessels}) c_{aHeart} \end{matrix}\right)/V_{aHeart}$ | (4a) |
| --- | --- |

where the first group of terms represents the recirculation in arterial blood flow for cytokines

| $\frac{dc_{vHeart}}{dt}= \left(\begin{matrix} ( Q_{liver}- L_{liver}) c_{liver}+( Q_{Torso}- L_{Torso}) c_{Torso} \\ +( Q_{upper body}- L_{upper body}) c_{upper body} \\ +( Q_{lower body}- L_{lower body}) c_{lower body} \\ +( Q_{cardic vessels}- L_{cardic vessels}) c_{cardic vessels} \\ +( Q_{brain}- L_{brain}) c_{brain} \\ +( Q_{kidney}- L_{kidney}) c_{kidney} \\ - (Q_{Hlung}+Q_{Tlung})c_{vHeart} \end{matrix}\right)/V_{vHeart}$ | (4b) |
| --- | --- |

where the first group of terms represents the recirculation in venous blood flow for cytokines

- 1. Liver

The liver compartment includes the hepatic portal vein from G.I. and spleen, and the hepatic artery to transport free viruses, antibody, and micro-thrombosis.

- - 1. Free virus in liver vessels: hepatic portal vein from G.I. and spleen, and hepatic artery

| $\frac{dv_{liver}}{dt}= \left(\begin{matrix} (Q_{G.I.}- L_{G.I.}) v_{G.I.} + ( Q_{spleen}- L_{spleen}) v_{spleen} \\ + ( Q_{liver}- Q_{G.I.}-Q_{spleen}+L_{G.I.}+L_{spleen}) v_{aHeart} \\ -(Q_{liver}- L_{liver}) v_{liver} \\ - a'_{liver} v_{liver} \left[sACE2\right] V_{liver} \\ - a_{liver} v_{liver} \left[ACE2\right]_{liver} V_{liver} \\ +d_{liver} v_{liver}^{b} V_{liver} \\ -k{}_{d} v_{liver} V_{liver} \\ +k_{a} v_{liver}^{\mathrm{int}}V_{liver} \\ -k_{v} v_{liver} A_{liver} V_{liver} \end{matrix}\right)/V_{liver}$ | (5a) |
| --- | --- |

where the first group of terms represents the recirculation in liver, $a_{i}$and $a'_{i}$ are respectively the attachment rates of free virus to ACE2 and sACE2, *d* is detachment rate of bound virus from ACE2, $k{}_{d}$is inactivating rate of the virus $k_{a}$is the rate of virus release from the cell and the last term describes the effect of the antibodies on the virus reproduction

- - 1. Virus bound to liver vessel wall

| $\frac{dv_{liver}^{b}}{dt}= \left(\begin{matrix} a_{liver} v_{liver} \left[ACE2\right]_{liver} V_{liver} \\ -d_{liver} v_{liver}^{b} V_{liver} \\ -k{}_{d} v_{liver}^{b} V_{liver} \\ -k_{\mathrm{int}} v_{liver}^{b} V_{liver} \end{matrix}\right)/V_{liver}$ | (5b) |
| --- | --- |

where $a_{i}$are the attachment rates of free virus to ACE2, *d* is the detachment rate of bound virus from ACE2, $k{}_{d}$is the inactivating rate of the virus and $k_{\mathrm{int}}$ is the rate of bound virus internalization

- - 1. Virus internalized into liver vessel endothelium

| $\frac{dv_{liver}^{\mathrm{int}}}{dt}= \left(\begin{matrix} p_{liver} k_{avp} v_{liver}^{b} \\ +k_{\mathrm{int}} v_{liver}^{b} V_{liver} \\ -k_{a} v_{liver}^{\mathrm{int}}V_{liver} \end{matrix}\right)/V_{liver}$ | (5c) |
| --- | --- |

where the first term describes replication, the second the internalization of the bound virus and the third term describes the viral particles that exit the cell.

- - 1. Micro-thrombus formation and transport in liver

|  | (5d) |
| --- | --- |

where the first group of terms represents the recirculation in liver, $k_{acg}$, $k'_{acg}$, $k''_{acg}$and $k'''_{acg}$ are the micro-thrombus-inhibiting coefficients of anti-virus drugs, anti-coagulation drugs for virus-ACE-2-induced coagulation, anti-coagulation drugs for IL6-induced coagulation,anti-coagulation drugs for cytokine storm-induced coagulation, $dm_{i}$ is the attachment rate of micro-thrombus to vessels. $\varepsilon_{i}$ is the dissolution of micro-thrombi due to thrombolysis.

- - 1. Micro-thrombus accumulation in liver

| $\frac{dT_{liver}^{b}}{dt}= \left( dm_{liver} T_{liver} V_{liver} -\varepsilon_{liver} T_{liver}^{b} V_{liver}\right)/V_{liver}$ | (5e) |
| --- | --- |

$dm_{i}$ is the attachment rate of micro-thrombus to vessels and $\varepsilon_{i}$ is the dissolution of micro-thrombi due to thrombolysis.

- - 1. ACE2 density of liver

| $\frac{d\left[ACE2\right]_{liver}}{dt}=d_{liver} v_{liver}^{b} - a_{liver} v_{liver}\left[ACE2\right]_{liver}$ | (5f) |
| --- | --- |

where the first term is the detachment rate of bound virus from ACE2 and the second term is the binding of the virus on the ACE2 receptor.

- - 1. Antibody transport in liver

| $\frac{dA_{liver}}{dt}= \left(\begin{matrix} (Q_{G.I.}- L_{G.I.}) A_{G.I.} + ( Q_{spleen}- L_{spleen}) A_{spleen} \\ + ( Q_{liver}- Q_{G.I.}-Q_{spleen}+L_{G.I.}+L_{spleen}) A_{aHeart} \\ -(Q_{liver}- L_{liver}) A_{liver} \\ - k_{A,v} v_{liver} A_{liver} V_{liver} \\ - k_{A,IEC} IEC_{liver} A_{liver} V_{liver} \\ - k_{a} A_{liver} V_{liver} \end{matrix}\right)/V_{liver}$ | (5g) |
| --- | --- |

where the first group of terms represents the recirculation in liver for antibody and the following terms describes the effect of the antibodies on the virus reproduction and the infected endothelial cell interaction and the last term describes the degradation rate of antibodies

- - 1. Cytokine transport in liver

| $\frac{dc_{liver}}{dt}= \left(\begin{matrix} (Q_{G.I.}- L_{G.I.}) c_{G.I.} + ( Q_{spleen}- L_{spleen}) c_{spleen} \\ + ( Q_{liver}- Q_{G.I.}-Q_{spleen}+L_{G.I.}+L_{spleen}) c_{aHeart} \\ -(Q_{liver}- L_{liver}) c_{liver} \\ + k_{c,NK} (NK) V_{liver}+ k_{c,\mathrm{DC}} (\mathrm{DC}^{*}) V_{liver} \\ + k_{c,\mathrm{Th}} (Th^{E}) V_{liver}+ k_{c,N} (N) V_{liver} \\ + k_{c,\mathrm{M}1} (M_{1}) V_{liver}+ k_{c,IEC}( IEC_{liver}) V_{liver} \\ +K_{c-IL6} [IL6-sIL6R](K_{c-EC}EC_{lung}) V_{liver} \\ - k_{c,\mathrm{Treg}} (T_{reg}) V_{liver}-d_{s} c_{liver} V_{liver} \end{matrix}\right)/V_{liver}$ | (5h) |
| --- | --- |

where the first group of terms represents the recirculation in liver for antibodies and the following terms describes the production of cytokines by the natural killer, antigen-presenting, effector CD4+ T cells, neutrophils, macrophages, infected endothelial cells, infected epithelial cells, the degradation rate of cytokines by Mas receptor, AT2 receptor and regulatory T cells, respectively, and the last term describes the degradation rate of cytokines

- - 1. Healthy endothelial cells of liver vessels

|  | (5i) |
| --- | --- |

Where the first term describes the overall (“effective”) proliferation/death of endothelial cells and the second term is the conversion of healthy endothelial cells to infected endothelial cells by the internalized virus, and the last term is the conversion of healthy endothelial cells to infected endothelial cells by neutrophils and cytokines

- - 1. Infected endothelial cells of liver vessels

|  | (5j) |
| --- | --- |

Where the first term is the conversion of healthy endothelial cells to infected endothelial cells by the internalized virus, and the second term is the conversion of healthy endothelial cells to infected endothelial cells by neutrophils and cytokines, and the last terms describe the overall (“effective”) death of infected endothelial cells.

- 1. For the i^th^ organ/tissue, where i = spleen, G.I., upper body, lower body, torso and cardiac vessels

All these compartments have upstream blood flow coming from the left ventricle of heart and downstream blood flow going back to the right ventricle of heart to allow both free viruses and micro-thrombosis to circulate in the whole body.

- - 1. Free virus in the vessels of i^th^ organ

| $\frac{dv_{i}}{dt}= \left(\begin{matrix} Q_{i} v_{aHeart}-(Q_{i}- L_{i}) v_{i} \\ - a'_{i} v_{i} \left[sACE2\right] V_{i} \\ - a_{i} v_{i} \left[ACE2\right]_{i} V_{i} \\ +d_{i} v_{i}^{b} V_{i} \\ -k{}_{d} v_{i} V_{i} \\ +k_{a} v_{i}^{\mathrm{int}} V_{i} \\ -k_{v} v_{i} A_{i} V_{i} \end{matrix}\right)/V_{i}$ | (6a) |
| --- | --- |

where the first group of terms represents the recirculation in i^th^ organ, $a_{i}$and $a'_{i}$ are respectively the attachment rates of free virus to ACE2 and sACE2, *d* is the detachment rate of bound virus from ACE2, $k{}_{d}$is the inactivating rate of the virus $k_{a}$is the rate of virus released from the cell and the last term describes the effect of the antibodies on the virus reproduction

- - 1. Bound virus on vessel wall of i^th^ organ

| $\frac{dv_{i}^{b}}{dt}= \left(\begin{matrix} a_{i} v_{i} \left[ACE2\right]_{i} V_{i} \\ -d_{i} v_{i}^{b} V_{i} \\ -k{}_{d} v_{i}^{b} V_{i} \\ -k_{\mathrm{int}} v_{i}^{b} V_{i} \end{matrix}\right)/V_{i}$ | (6b) |
| --- | --- |

where $a_{i}$are the attachment rates of free virus to ACE2, *d* is the detachment rate of bound virus from ACE2, $k{}_{d}$is the inactivating rate of the virus and $k_{\mathrm{int}}$ is the rate of bound virus internalization.

- - 1. Internalized virus in endothelium of i^th^ organ

| $\frac{dv_{i}^{\mathrm{int}}}{dt}= \left(\begin{matrix} p_{i} k_{avp} v_{i}^{b} \\ +k_{\mathrm{int}} v_{i}^{b} V_{i} \\ -k_{a} v_{i}^{\mathrm{int}} V_{i} \end{matrix}\right)/V_{i}$ | (6c) |
| --- | --- |

where the first term describes replication, the second the internalization of the bound virus and the third term describes the viral particles that exit the cell.

- - 1. Micro-thrombus formation and transport in i^th^ organ

|  | (6d) |
| --- | --- |

where the first group of terms represents the recirculation in i^th^ organ , $k_{acg}$, $k'_{acg}$, $k''_{acg}$and $k'''_{acg}$ are the micro-thrombus-inhibiting coefficients of anti-virus drugs, anti-coagulation drugs for virus-ACE-2-induced coagulation, anti-coagulation drugs for IL6-induced coagulation,anti-coagulation drugs for cytokine storm-induced coagulation, $dm_{i}$ is the attachment rate of micro-thrombus to vessels. $\varepsilon_{i}$ is the dissolution of micro-thrombi due to thrombolysis.

- - 1. Micro-thrombus accumulation in i^th^ organ

| $\frac{dT_{i}^{b}}{dt}= \left( dm_{i} T_{i} V_{i} -\varepsilon_{i} T_{i}^{b} V_{i}\right)/V_{i}$ | (6e) |
| --- | --- |

$dm_{i}$ is the attachment rate of micro-thrombus to vessels. $\varepsilon_{i}$ is the dissolution of micro-thrombi due to thrombolysis.

- - 1. ACE2 density of i^th^ organ

| $\frac{d\left[ACE2\right]_{i}}{dt}=d_{i} v_{i}^{b} - a_{i} v_{i}\left[ACE2\right]_{i}$ | (6f) |
| --- | --- |

where the first term is the detachment rate of bound virus from ACE2 and the second term is the binding of the virus on the ACE2 receptor.

- - 1. Antibody transport in i^th^ organ

| $\frac{dA_{i}}{dt}= \left(\begin{matrix} Q_{i} A_{aHeart}-(Q_{i}- L_{i}) A_{i} \\ - k_{A,v} v_{i} A_{i} V_{i} \\ - k_{A,IEC} IEC_{i} A_{i} V_{i} \\ - k_{a} A_{i} V_{i} \end{matrix}\right)/V_{i}$ | (6g) |
| --- | --- |

where the first group of terms represents the recirculation in ith organ for antibody and the following terms describe the effect of the antibodies on the virus reproduction and the infected endothelial cell interaction and the last term describes the degradation rate of antibodies

- - 1. cytokine transport in i^th^ organ

| $\frac{dc_{i}}{dt}= \left(\begin{matrix} Q_{_{i}} c_{vHeart}-(Q_{i}- L_{i}) c_{i} \\ + k_{c,NK} (NK) V_{i}+ k_{c,\mathrm{DC}} (\mathrm{DC}^{*}) V_{i} \\ + k_{c,\mathrm{Th}} (Th^{E}) V_{i}+ k_{c,N} (N) V_{i} \\ + k_{c,\mathrm{M}1} (M_{1}) V_{i}+ k_{c,IEC}( IEC_{i}+In_{i}) V_{i} \\ +K_{AT1R} [AT1R-ANGII] V_{i} \\ +K_{c-IL6} [IL6-sIL6R](K_{c-EC}EC_{i}+K_{c-H}H_{i})V_{i} \\ -K_{AT1R-MAsR} [MAsR] V_{i}-K_{AT1R-AT2R}[AT2R] V_{i} \\ - k_{c,\mathrm{Treg}} (T_{reg}) V_{i}-d_{s} c_{i} V_{i} \end{matrix}\right)/V_{i}$ | (6h) |
| --- | --- |

where the first group of terms represents the recirculation in ith organ for antibodies and the following terms describe the production of cytokines by the natural killer, antigen-presenting, effector CD4+ T cells, neutrophils, macrophages, infected endothelial cells, infected epithelial cells, the degradation rate of cytokines by Mas receptor, AT2 receptor and regulatory T cells, respectively, and the last term describes the degradation rate of cytokines

- - 1. Healthy endothelial cells of liver vessels

|  | (6i) |
| --- | --- |

Where the first term describes the overall (“effective”) proliferation/death of endothelial cells and the second term is the conversion of healthy endothelial cells to infected endothelial cells by internalized virus, and the last term is the conversion of healthy endothelial cells to infected endothelial cells by neutrophils and cytokines.

- - 1. Infected endothelial cells of liver vessels

|  | (6j) |
| --- | --- |

Where the first term is the conversion of healthy endothelial cells to infected endothelial cells by internalized virus, and the second term is the conversion of healthy endothelial cells to infected endothelial cells by neutrophils and cytokines, and the last terms describe the overall (“effective”) death of infected endothelial cells.

- 1. Lung

The lung compartment has upstream blood flow coming from heart and downstream blood flow going back to heart to transport both free viruses and micro-thrombi. In this study, the lung compartment is divided into two different parts: normal healthy lung (*Hlung*), tumor part of lung (*Tlung*). Free, bound and internalized virus concentrations in the lung are calculated using the microscale model of the lung, Eqs. 33-35. ACE2 concentration of the lung is calculated by Eq. 32 of the microscale model of lung. The microthrombus dynamics are:

Normal part of Lung :

- - 1. Micro-thrombus formation and transport in healthy regions of lung

|  | (7a) |
| --- | --- |

where the first group of terms represents the recirculation in healthy regions of lung , $k_{acg}$, $k'_{acg}$, $k''_{acg}$and $k'''_{acg}$ are the micro-thrombus-inhibiting coefficients of anti-virus drugs, anti-coagulation drugs for virus-ACE-2-induced coagulation, anti-coagulation drugs for IL6-induced coagulation,anti-coagulation drugs for cytokine storm-induced coagulation and NETs, $\varepsilon_{i}$ is the dissolution of micro-thrombi due to thrombolysis, $dm_{i}$ is the attachment rate of micro-thrombus to vessels.

- - 1. Micro-thrombus accumulation in lung

| $\frac{dT_{\text{Hlung}}^{b}}{dt}= \left( dm_{\text{lung}} T_{\text{Hlung}} V_{\text{Hlung}} -\varepsilon_{\text{lung}} T_{\text{Hlung}}^{b} V_{\text{Hlung}}\right)/V_{\text{Hlung}}$ | (7b) |
| --- | --- |

$dm_{i}$ is the attachment rate of micro-thrombus to vessels. $\varepsilon_{i}$ is the dissolution of micro-thrombi due to thrombolysis.

- - 1. Antibody transport in lung

|  | (7c) |
| --- | --- |

where the first group of terms represents the recirculation in healthy regions of lung for antibodies, the following terms describe the antibodies production by long and short-lived plasma respectively, the effect of the antibodies on the virus reproduction and the infected endothelial and epithelial cell interaction and the last term describes the degradation rate of antibodies

- - 1. Cytokine transport in lung

| $\frac{dc_{\text{Hlung}}}{dt}= \left(\begin{matrix} Q_{Hlung} c_{vHeart}-(Q_{Hlung}- L_{Hlung}) c_{Hlung} \\ + k_{c,NK} (NK) V_{Hlung}+ k_{c,\mathrm{DC}} (\mathrm{DC}^{*}) V_{Hlung} \\ + k_{c,\mathrm{Th}} (Th^{E}) V_{Hlung}+ k_{c,N} (N) V_{Hlung} \\ + k_{c,\mathrm{M}1} (M_{1}) V_{Hlung}+ k_{c,IEC}( IEC_{Hlung}+In_{Hlung}) V_{Hlung} \\ +K_{AT1R} [AT1R-ANGII] V_{Hlung} \\ +K_{c-IL6} [IL6-sIL6R](K_{c-EC}EC_{Hlung}+K_{c-H}H_{Hlung})V_{Hlung} \\ -K_{AT1R-MAsR} [MAsR] V_{Hlung}-K_{AT1R-AT2R}[AT2R] V_{Hlung} \\ - k_{c,\mathrm{Treg}} (T_{reg}) V_{Hlung}-d_{s} c_{Hlung} V_{Hlung} \end{matrix}\right)/V_{Hlung}$ | (7d) |
| --- | --- |

where the first group of terms represents the recirculation in healthy regions of lung for antibodies and the following terms describe the production of cytokines by the natural killer, antigen-presenting, effector CD4+ T cells, neutrophils, macrophages, infected endothelial cells, infected epithelial cells, the degradation rate of cytokines by MAs receptor, AT2 receptor and regulatory T cells, respectively, and the last term describes the degradation rate of cytokines

- - 1. Healthy endothelial cells of lung vessels

|  | (7e) |
| --- | --- |

Where the first term describes the overall (“effective”) proliferation/death of endothelial cells and the second term is the conversion of healthy endothelial cells to infected endothelial cells by internalized virus, and the last term is the conversion of healthy endothelial cells to infected endothelial cells by neutrophils and cytokines.

- - 1. Infected endothelial cells of lung vessels

|  | (7f) |
| --- | --- |

Where the first term is the conversion of healthy endothelial cells to infected endothelial cells by internalized virus, and the second term is the conversion of healthy endothelial cells to infected endothelial cells by neutrophils and cytokines, and the last terms describe the overall (“effective”) death of infected endothelial cells.

- - 1. Healthy epithelial cells of lung vessels

|  | (7g) |
| --- | --- |

Where the first term describes the overall (“effective”) proliferation/death of epithelial cells and the second term is the conversion of healthy epithelial cells to infected epithelial cells by internalized virus, and the last term is the conversion of healthy epithelial cells to infected endothelial cells by neutrophils and cytokines.

- - 1. Infected epithelial cells of lung vessels

|  | (7h) |
| --- | --- |

Where the first term is the conversion of healthy endothelial cells to infected epithelial cells by internalized virus, and the second term is the conversion of healthy epithelial cells to infected endothelial cells by neutrophils and cytokines, and the last terms describe the overall (“effective”) death of infected epithelial cells.

Tumor part of lung:

- - 1. Micro-thrombus formation and transport in lung tumor

|  | (8a) |
| --- | --- |

where the first group of terms represent the recirculation in lung tumor , $k_{acg}$, $k'_{acg}$, $k''_{acg}$and $k'''_{acg}$ are the micro-thrombus-inhibiting coefficients of anti-virus drugs, anti-coagulation drugs for virus-ACE-2-induced coagulation, anti-coagulation drugs for IL6-induced coagulation,anti-coagulation drugs for cytokine storm-induced coagulation and NETs, $\varepsilon_{i}$ is the dissolution of micro-thrombi due to thrombolysis, $dm_{i}$ is the attachment rate of micro-thrombus to vessels.

- - 1. Micro-thrombus accumulation in lung tumor

| $\frac{dT_{\text{Tlung}}^{b}}{dt}= \left( dm_{\text{lung}} T_{\text{Tlung}} V_{\text{Tlung}} -\varepsilon_{\text{lung}} T_{\text{Tlung}}^{b} V_{\text{Tlung}}\right)/V_{\text{Tlung}}$ | (8b) |
| --- | --- |

$dm_{i}$ is the attachment rate of micro-thrombus to vessels. $\varepsilon_{i}$ is the dissolution of micro-thrombi due to thrombolysis.

- - 1. Antibody transport in lung tumor

|  | (8c) |
| --- | --- |

where the first group of terms represents the recirculation in lung tumor for antibodies, the following terms describe the antibodies production by long and short-lived plasma respectively, the effect of the antibodies on the virus reproduction and the infected endothelial and epithelial cell interaction and the last term describes the degradation rate of antibodies.

- - 1. cytokine transport in the tumor part of lung

| $\frac{dc_{Tlung}}{dt}= \left(\begin{matrix} Q_{Tlung} c_{vHeart}-(Q_{Tlung}- L_{Tlung}) c_{Tlung} \\ + k_{c,NK} (NK) V_{Tlung}+ k_{c,\mathrm{DC}} (\mathrm{DC}^{*}) V_{Tlung} \\ + k_{c,\mathrm{Th}} (Th^{E}) V_{Tlung}+ k_{c,N} (N) V_{Tlung} \\ + k_{c,\mathrm{M}1} (M_{1}) V_{Tlung}+ k_{c,IEC}( IEC_{Tlung}+In_{Tlung}) V_{Tlung} \\ +K_{AT1R} [AT1R-ANGII] V_{Tlung} \\ +K_{c-IL6} [IL6-sIL6R](K_{c-EC}EC_{Tlung}+K_{c-H}H_{Tlung})V_{Tlung} \\ -K_{AT1R-MAsR} [MAsR] V_{Tlung}-K_{AT1R-AT2R}[AT2R] V_{Tlung} \\ - k_{c,\mathrm{Treg}} (T_{reg}) V_{Tlung}-d_{s} c_{Tlung} V_{Tlung} \end{matrix}\right)/V_{Tlung}$ | (8d) |
| --- | --- |

where the first group of terms represents the recirculation in lung tumor for antibodies and the following terms describe the production of cytokines by the natural killer, antigen-presenting, effector CD4+ T cells, neutrophils, macrophages, infected endothelial cells, infected epithelial cells, the degradation rate of cytokines by MAs receptor, AT2 receptor and regulatory T cells, respectively, and the last term describes the degradation rate of cytokines.

- - 1. Healthy endothelial cells of the vessels of lung tumor

|  | (8e) |
| --- | --- |

Where the first term describes the overall (“effective”) proliferation/death of endothelial cells and the second term is the conversion of healthy endothelial cells to infected endothelial cells by internalized virus, and the last term is the conversion of healthy endothelial cells to infected endothelial cells by neutrophils and cytokines.

- - 1. Infected endothelial cells of the vessels of lung tumor

|  | (8f) |
| --- | --- |

Where the first term is the conversion of healthy endothelial cells to infected endothelial cells by internalized virus, and the second term is the conversion of healthy endothelial cells to infected endothelial cells by neutrophils and cytokines, and the last terms describe the overall (“effective”) death of infected endothelial cells.

- - 1. Healthy epithelial cells of the vessels of lung tumor

|  | (8g) |
| --- | --- |

Where the first term describes the overall (“effective”) proliferation/death of epithelial cells and the second term is the conversion of healthy epithelial cells to infected epithelial cells by internalized virus, and the last term is the conversion of healthy epithelial cells to infected endothelial cells by neutrophils and cytokines.

- - 1. Infected epithelial cells of the vessels of lung tumor

|  | (8h) |
| --- | --- |

Where the first term is the conversion of healthy endothelial cells to infected epithelial cells by internalized virus, and the second term is the conversion of healthy epithelial cells to infected endothelial cells by neutrophils and cytokines, and the last terms describe the overall (“effective”) death of infected epithelial cells.

- 1. Mass conservation

| $\begin{matrix} \text{Q}_{\text{Hlung}}\text{+Q}_{\text{Tlung}}\text{=L}_{\text{Hlung}}\text{+L}_{\text{Tlung}}\text{+Q}_{liver}\text{+L}_{G.I.}\text{+L}_{\text{spleen}}\text{+Q}_{\text{kidney}} \\ \text{ } \text{+Q}_{\text{Torso}}\text{+Q}_{lower body}\text{+Q}_{upper body}\text{+Q}_{brain}\text{+Q}_{cardiac vessels} \end{matrix}$ | (9) |
| --- | --- |

**Simplifying Assumptions:**

**T_E_,** Ma, N, a (Anti-inflammatory cytokines), , IL6, IF, T^M^ are uniform.

Antibodies are released in lung and then circulate. There is a production term just for lung. But we could assume uniform short- and Long-lived B cells and add production terms to each compartment.

1. Microscale Lung
   1. Equations describing the Renin-Angiotensin system

The reaction of angiotensinogen (AGT) is governed by Eq. 7,[9]


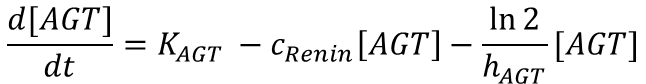
 (10)

The rate of change of angiotensinogen depends on its production (K_AGT_); production of ANG I catalyzed by renin is assumed to follow first order kinetics, and thus PRA = c_Renin_[AGT], degradation of AGT is considered to exhibit first-order kinetics in terms of its half-life h_AGT_.

The mass balance for Renin is [9]


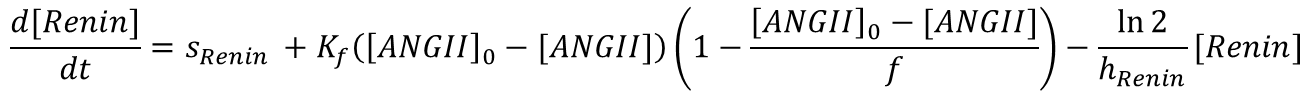
 (11)

The first term, s_Renin_ , accounts for a constant source of renin from the kidney; the second term is the influence of ANG II negative feedback on renin production, [ANG II]_0_ is the initial concentration of ANG II, k_f_ and f are parameters for the feedback, and h_Renin_ is the half-life for the degradation of renin.

(12)

The renin source term is computed at steady state, where [Renin]_0_ is the initial concentration of renin.

The mass balance for ANG I is[9]

$\frac{d[ANGI]}{dt}=c_{Renin}\left[ AGT \right]+K_{Renin}\left( {\left[ Renin \right]-\left[ Renin \right]}_{0} \right)-K_{ACE}\left[ ANGI \right]-K_{NEP}ANGI-K_{ACE2-ANGI}^{on}\left[ ANGI \right]\left[ ACE2 \right]+K_{ACE2-ANGI}^{off}\left[ ACE2-ANGI \right]-\frac{\ln2}{h_{ANGI}}[ANGI]$ (13)

where the first term represents the glucose-dependent renin-catalyzed contribution to the production of ANG I from AGT, the second term represents the change to ANG I synthesis from AGT due to the feedback of ANG II on renin with rate constant K_Renin_, the third term is the ACE-catalyzed conversion of ANG I to ANG II and has a glucose-dependent rate parameter K_ACE_, the fourth term is the consumption of ANG I to form ANG-(1-7) with the glucose-independent rate parameters K_NEP_, the following two terms describe the binding/unbinding of ANG I on the ACE 2 receptors, and h_ANG I_ is the half-life for the degradation of ANG I.

The mass balance for the free ANG II is[9]

(14)

where the first term is the production of ANG II in the presence of ACE, the following two terms

The mass balance for ANGII bound to AT1 receptor is

(15)

where the first two terms describe the binding/unbinding of ANG II on the AT1 receptors and hAT1R-ANGII is the degradation half-life.

The mass balance for ANGII bound to AT2 receptor is[10]

(16)

where the first two terms describe the binding/unbinding of ANG II on the AT2 receptor and has a degradation half-life h_AT2R-ANGII_.

The mass balance for ANG(1-7) is[10]

(17)

where the first three terms are the production of ANG(1-7) by ANGI, ANGII bound to ACE2, and by ANG(1-9), the following two terms describe the binding/unbinding of ANG 1-7 to the MAs receptors and h_ANG 1-7_ is the half-life for degradation of ANG 1-7.

The mass balance for ANG(1-7) bound to Mas receptor is

(18)

where the first two terms describe the binding/unbinding of ANG 1-7 on the MAs receptor and has a degradation half-life of h_MasR-ANG1-7_.

The mass balance of ANG (1-9) is[10]

(19)

where the first term is the production of ANG(1-9) by ANGI bound to the ACE2 and h_ANG 1-9_ is the half-life for degradation of ANG 1-9.

The mass balance term of ANGIII is[10]

(20)

where the first term is the production of ANGIII by ANG II, the second term describes the production of ANGIV by ANGIII and h_ANG III_ is the half-life for degradation of ANG III.

The mass balance for ANGIV is[11]

(21)

where the first term is the production of ANG IV by ANG III, the following two terms describe the binding/unbinding of ANG IV on the AT4 receptor and h_ANGIV_ the half-life for degradation of ANG IV.

The mass balance for ANGIV bound to AT4 receptor is

(22)

where the first two terms describe the binding/unbinding of ANG IV on the AT4 receptors and has a degradation half-life h_AT4R-ANGIV_.

The mass balance for ANGI bound to ACE2 is

(23)

where the first two terms describe the binding/unbinding of ANGI on the ACE2 and the last term describes the degradation of ANGI bound to ACE2.

Mass balance for ANGII bound to ACE2:

(24)

${\frac{d\left[ ACE2-ANGII \right]}{dt}=K}_{ACE2-ANGII}^{on}\left[ ANGII \right]\left[ ACE2 \right]- K_{ACE2-ANGII}^{off}\left[ ACE2-ANGII \right]-K_{ACE2} \left[ ACE2-ANGII \right]$

where the first two terms describe the binding/unbinding of ANGII on the ACE2 and the last term describes the degradation of the ANGII bound to ACE2.

The mass balance for AT1 receptor is

(25)

where the first term represents the source term for AT1 receptor, the following two terms describe the binding/unbinding of ANG II on the AT1 receptor, and the last term describes the degradation of the AT1 receptor.

The mass balance for AT2 receptor is

(26)

where the first term represents the source term for AT2 receptor, the following two terms describe the binding/unbinding of ANG II on the AT2 receptors, and the last term describes the degradation of the AT2 receptor.

The mass balance for MAs receptor is

(27)

where the first term represents the source term for MAs receptor, the following two terms describe the binding/unbinding of ANG 1-7 on the MAs receptors, and the last term describes the degradation of the MAs receptor.

The mass balance for AT4 receptor is

(28)

where the first term represents the source term for AT4 receptor, the following two terms describe the binding/unbinding of ANG IV on the AT4 receptors, and the last term describes the degradation of the AT4 receptor.

- 1. Mass Balance Equations for IL6, IL6 receptor, soluble IL6R, binding of IL6 to IL6R and sIL6R and production of VEGF

The reaction rate of the IL-6 reads

| $\frac{d\left[ IL6 \right]}{dt}=K_{IL6-Nk}Nk+K_{IL6-DC}{DC}^{*}+K_{IL6-TE}\left( {Th}^{E}+T^{E} \right)+K_{IL6-M1}M_{1}-\gamma_{IL6}\left[ IL6 \right]$ $-K_{IL6}^{on}\left[ IL6 \right]\left[ IL6R \right]+K_{IL6}^{off}\left[ IL6R-IL6 \right]{-K_{sIL6R}^{on}\left[ sIL6R \right]\left[ IL6 \right]+K}_{sIL6R}^{off}\left[ sIL6R-IL6 \right]-K_{sIL6R}^{on}\left[ sIL6R \right]\left[ IL6 \right]$ | (29) |
| --- | --- |

where the first four terms are the production of IL-6 by the natural killer, activated dendritic, and effector T cells, and the type 1 macrophages, respectively. The fifth term is the degradation of the IL-6. The rest terms describe the binding/unbinding of IL-6 to the IL-6 receptor and soluble IL-6 receptor.

The reaction rate of the IL-6 receptor reads

| $\frac{d[IL6R]}{dt}=S_{IL6R}-K_{IL6}^{on}\left[ IL6 \right]\left[ IL6R \right]+K_{IL6}^{off}\left[ IL6R-IL6 \right]-K_{sIL6R}\left[ IL6R \right]-d_{IL6R}[IL6R]$ | (30) |
| --- | --- |

where the first term represents the source term for IL-6 receptor, the following two terms describe the binding/unbinding of IL-6 to the IL-6 receptors and the last terms are the transformation to soluble IL-6 receptors and the degradation of IL-6 receptors respectively.

The reaction rate of the soluble IL-6 receptor reads

| $\frac{d[sIL6R]}{dt}=K_{sIL6R}\left[ IL6R \right]+K_{sIL6R}^{off}[sIL6R-IL6]-K_{sIL6R}^{on}\left[ sIL6R \right]\left[ IL6 \right]$ | (31) |
| --- | --- |

where the first term is the transformation of IL-6 receptor to the soluble IL-6 receptor and the last terms describe the binding/unbinding of IL6 to the soluble IL-6 receptors.

The reaction rate of the IL-6 - IL-6 receptor complex reads

| $\frac{d[IL6R-IL6]}{dt}=K_{IL6}^{on}\left[ IL6 \right]\left[ IL6R \right]-K_{IL6}^{off}[IL6R-IL6]-\frac{\ln2}{h_{IL6R}}[IL6R-IL6]$ | (32) |
| --- | --- |

where the first two terms describe the binding/unbinding of IL-6 to IL-6 receptor and the last term is the degradation of IL-6-IL-6 receptor complex (with a half-life $h_{IL6R}$)

The reaction rate of the IL-6-soluble receptor - IL-6 complex reads

| $\frac{d\left[ sIL6R-IL6 \right]}{dt}=K_{sIL6R}^{on}\left[ sIL6R \right][IL6]{-K}_{sIL6R}^{off}\left[ sIL6R-IL6 \right]$ | (33) |
| --- | --- |

where the two terms describe the binding/unbinding of IL-6 to the soluble IL-6 receptor.

The mass balance for VEGF

$$\frac{d\left[ VEGF \right]}{dt}=\nabla\cdot\left( D_{VEGF}\nabla\left[ VEGF \right] \right)+K_{T}G_{a}\left( c_{ox} \right)T+K_{T_{reg}}G_{a}\left( c_{ox} \right)T_{reg}+K_{VEGF}\left[ sIL6R-IL6 \right]-d_{VEGF}\left[ VEGF \right]+\gamma_{VEGF}\left( 100-SPO2 \right)$$

VEGF is assumed to be produced by cancer cells and *T_regs_* and its production is enhanced under hypoxic conditions as described by the oxygen tension term *G_a._* The following term describes the production of VEGF by IL-6 bound on the soluble IL-6 receptor, the following term is the degradation rate of the VEGF and the last term describes the production of VEGF by hypoxia

(34)

| *G_a_($\hat{c_{ox}}$)=* | *3$\hat{c_{ox}}$ for 0<$\hat{c_{ox}}$<0.5 (hypoxia)*  *2 -$\hat{c_{ox}}$ for 0.5<$\hat{c_{ox}}$<1 (normoxia)* $\hat{c_{ox}}$ *for 1<$\hat{c_{ox}}$ (hyperoxia)* |  |
| --- | --- | --- |
|  |  |  |

- 1. Model equations for virus infection and immune cells activation

The mass balance for soluble ACE2 receptor

(35)

$$\frac{d[sACE2]}{dt}=- K_{sACE2}\left[ sACE2 \right]\left[ V \right]-\sum_{i} K_{sACE2}\left[ sACE2 \right]\left[ v_{i} \right]+K_{Adam17}[ACE2]-d_{sACE2}\left[ sACE2 \right]$$

where the first term is the binding of soluble ACE2 receptor to the virus, the third term is internalization everywhere, and the following term is the production of soluble ACE2 receptor by ACE2 receptor interaction through Adam17 and the last term is the degradation rate of the soluble ACE2 receptor.

The mass balance of ACE2 receptor is

(36)

$\frac{d[ACE2]}{dt}=S_{ACE2}(\left[ EC \right]+H)-K_{ACE2-Virus}^{on}\left[ v \right]\left[ ACE2 \right]+K_{ACE2-Virus}^{off}\left[ v_{b} \right]-K_{Adam17}[ACE2]-K_{ACE2-ANGI}^{on}\left[ ANGI \right]\left[ ACE2 \right]+K_{ACE2-ANGI}^{off}\left[ ACE2-ANGI \right]-K_{ACE2-ANGII}^{on}\left[ ANGII \right]\left[ ACE2 \right]+ K_{ACE2-ANGII}^{off}[ACE2-ANGII]$

where the first term describes the production of ACE2 receptor by endothelial and epithelial cells, the second and third term describes the interaction with the virus, the fourth term describes the production of soluble ACE2 and the rest terms describe the interaction of ACE2 with ANGI and ANGII.

The virus can be in three states, the free virus that diffuses in the lung tissue, the bound virus on the epithelial cells of the lungs and the internalized virus. The equations for the three states of the virus are:

Free virus in normal Lung

(37)

Where the terms respectively describe convectional transport of free virus in lung, the binding of the virus to soluble ACE2, the binding of the virus to ACE2, the detachment of bound virus from ACE2, the inactivating of the virus, and the source of new viral particles from the release of the infected cells following cell death.

The effect of the antibodies on the virus reproduction is represented by the term [24](3).

Free virus in Tumor part of lung

(38)

Where the terms respectively describe convectional transport of free virus in tumor, the binding of the virus to soluble ACE2, the binding of the virus to ACE2, the detachment of bound virus from ACE2, the inactivating of the virus, and the source of new viral particles from the release of the infected cells following cell death.

The effect of the antibodies on the virus reproduction is represented by the term [24](3).

Bound virus in normal lung

(39)

where the first two terms describe the binding/unbinding of the virus on the ACE2 receptor, the third term is the degradation of the bound virus, the last term is the internalization of the bound virus into the cells.

Internalized virus in normal lung

(40)

where the first term describes replication, the second the internalization of the bound virus and the third term describes the viral particles that exit the cell.

Bound virus in tumor part of lung

(41)

where the first two terms describe the binding/unbinding of the virus on the ACE2 receptor, the third term is the degradation of the bound virus, the last term is the internalization of the bound virus into the cells.

Internalized virus in tumor part of lung

(42)

where the first term describes replication of virus in tumor region of lung, the second term is the internalization of the bound virus and the third term describes the viral particles that exit the tumor cell.

- 1. Interferon gamma ($\boldsymbol{IF}\boldsymbol{N}_{\boldsymbol{\gamma}}$)

The reaction rate of the interferon gamma reads

| $\frac{d{IFN}_{\gamma}}{dt}=k_{IFN_{\gamma} NK}NK+k_{IFN_{\gamma} {Th}^{E}}{Th}^{E}+k_{IFN_{\gamma} T^{E}}T^{E}+a_{IF} \left( IEC+In \right)+k_{IFN_{\gamma}}- \varepsilon_{IF} {IFN}_{\gamma}$ | (43) |
| --- | --- |

where the first five terms describe the production rate of the interferon gamma by the natural killer, effector CD4^+^ T, effector CD8^+^ T, infected endothelial, infected epithelial, and other cells, respectively. The last term describes the degradation rate of interferon gamma.

- 1. Anti-inflammatory cytokines

The reaction rate of the anti-inflammatory cytokines reads

| $\frac{da}{dt}=k_{a-N}N*\left( M_{a}+DC \right)+K_{Ang1-7}\left[ MAsR-{ANG}_{\left( 1-7 \right)} \right]+K_{ant-T_{reg}}T_{reg}+K_{ant-Ma}M_{a}-\gamma_{a}a$ | (44) |
| --- | --- |

where the first term describes the production of anti-inflammatory cytokines by type 2 macrophages and dendritic cells after phagocytosis of apoptotic neutrophils. The other three terms are the production by ANG(1-7)-MasR complex, regulatory T cells and type 2 macrophages respectively. The last term is the degradation rate of anti-inflammatory cytokines.

- 1. Reactions of PDL-1, PD-1, and anti-PD-1

The reaction of the PDL-1 reads

| $\frac{d[PDL1]}{dt}=\left[ \left( \lambda_{H}-d_{H} \right)H+\left( \lambda_{EC}-d_{EC} \right)\left[ EC \right]+\left( \lambda_{T}-d_{T} \right)\hat{T} \right]\frac{\left[ PDL1 \right]}{H+\left[ EC \right]+\hat{T}}+h_{PDL1}T_{E}+h_{M2}M_{a}$ | (45) |
| --- | --- |

where the first term describes the production(*λ*_i_)/degradation(*d*_i_) of the PD1 ligand by healthy epithelial, healthy endothelial cells and tumor cells, the last two terms describe the production of the PD1 ligand by Effector (Activated) T cells and type 2 macrophages.

The reaction of the PD-1 reads

| $\frac{d\left[ PD1 \right]}{dt}=\left[ \left( \lambda_{TE}-d_{TE} \right)T_{E}+\left( \lambda_{TN}-d_{TN} \right)T_{N}+\left( \lambda_{N}-d_{N} \right)N+\left( \lambda_{NK}-d_{NK} \right)NK+\left( \lambda_{M2}-d_{M2} \right)M_{a} \right]\frac{\left[ PD1 \right]}{T_{E}+T_{N}+N+NK+M_{a}}-\mu_{PD1-aPD1}\left[ PD1 \right]\left[ anti-PD1 \right]$ | (46) |
| --- | --- |

where the first describes the production(*λ*_i_)/degradation(*d*_i_) of the PD1 by Effector (Activated) T cells, Naïve T cells, neutrophils, natural killer cells and macrophages. The last term describes the binding of the anti-PD1 to PD1.

The reaction of the PD-1 – PDL-1 complex reads

| $\frac{d\left[ PD1-PDL1 \right]}{dt}=a_{PL}\left[ PD1 \right]\left[ PDL1 \right]-d_{Q}\left[ PD1-PDL1 \right]$ | (47) |
| --- | --- |

where the first term describes the binding of PD1 to the PD1 ligand and the last term describes its degradation rate.

The reaction of the anti-PD-1 reads

| $\frac{d\left[ anti-PD1 \right]}{dt}=\gamma_{A}-\mu_{PD1-aPD1}\left[ PD1 \right]\left[ anti-PD1 \right]-d_{A}\left[ anti-PD1 \right]$ | (48) |
| --- | --- |

where the first term represents the source term of the anti-PD1, the second term describes the production of the anti-PD1 - PD1 complex and the last term describes its degradation rate.

- 1. Cells of immune system
     1. Neutrophils

The reaction rate of the neutrophils reads

| $\frac{dN}{dt}=\frac{\chi_{N} c}{1+a}+\chi_{N-IL6}\left[ IL6R-IL6 \right]-\gamma_{n}N*(1+\beta_{n}\frac{v}{v+1})$ | (49) |
| --- | --- |

Where the production rate of the neutrophils depends on cytokines (pro/anti-inflammatory) and IL-6 - IL-6R complex, and the last term describes their death rate.

- - 1. Neutrophils Extracellular Traps (NETs)

(50)

where the first term describes the production Neutrophil extracellular traps and the last term is their degradation rate.

- - 1. Immature dendritic cells (DC)

The reaction rate of the immature dendritic cells reads

|  | (51) |
| --- | --- |

where the first term describes the source term of DC and the second term is the death rate of DC, the rest term describes the conversion of dendritic cells to antigen presenting cells and the last term describes the degradation rate of DC by regulatory T cells.

- - 1. Antigen presenting cells APCs (*DC^*^*)

The reaction rate of the APCs reads

|  | (52) |
| --- | --- |

where the first two terms describe the conversion rates of dendritic cells to APCs, , the rest term describes the degradation rate of APCs by regulatory T cells and the last term describes the death rate of APCs.

- - 1. Naïve CD4^+^ T cells (*Th^N^*)

The reaction rate of the Naive CD4^+^ T cells reads

| $\frac{d{Th}^{N}}{dt}=S_{ThN}-h_{Th}{DC}^{*}{Th}^{N}\left[ \left( \frac{V_{proteins}}{1+V_{proteins}} \right)+\left( \frac{As}{1+As} \right) \right]\left( \frac{c}{1+c} \right)\left( \frac{{IFN}_{\gamma}}{K_{IF}+{IFN}_{\gamma}} \right) \frac{K_{T}}{K_{T}+\left[ PD1-PDL1 \right]}-d_{TN}{Th}^{N}-\frac{h_{Treg}DC {Th}^{N}a}{\left( K_{Th^{N} DC}+DC \right)\left( K_{DC Th^{N}}+{Th}^{N} \right)\left( K_{T_{reg} a}+a \right)}$ | (53) |
| --- | --- |

where the first term describes the source term of Naïve CD4^+^ T cells, the second term describes the activation rate of naive CD4^+^ T cells, the following term describes the death rate of naïve CD4^+^ T cells and the last term describes its transformation to regulatory T cells.

- - 1. Effector CD4^+^ T cells (*Th^E^*)

The reaction rate of the effector CD4^+^ T cells reads

| $\frac{d{Th}^{E}}{dt}=h_{Th} {DC}^{*}{Th}^{N}\left[ \left( \frac{V_{proteins}}{1+V_{proteins}} \right)+\left( \frac{As}{1+As} \right) \right]\left( \frac{c}{1+c} \right)\left( \frac{{IFN}_{\gamma}}{K_{IF}+{IF}_{\gamma}} \right) \frac{K_{T}}{K_{T}+\left[ PD1-PDL1 \right]}+\rho_{N}{Th}^{E}\left( \frac{As}{1+As} \right)\left( \frac{c}{1+c} \right)\left( \frac{{IFN}_{\gamma}}{K_{IF}+{IFN}_{\gamma}} \right) \frac{K_{T}}{K_{T}+\left[ PD1-PDL1 \right]}-\pi_{ThM}{Th}^{E}-\varepsilon{Th}^{E}\frac{\left[ PD1-PDL1 \right]}{AS}-\frac{k_{Treg_{1}}{Th}^{E}T_{reg}}{(1+{Th}^{E})(1+T_{reg})}$ | (54) |
| --- | --- |

where the first term describes the activation rate of naïve CD4^+^ T cells, the second term describes the proliferation rate of effector CD4^+^ T cells, the third term describes the conversion rate of effector CD4^+^ T cells to memory CD4^+^ T cells and the last two terms describe their death rates which depend on the PD1 – PDL1 complex and the regulatory T cells.

- - 1. Regulatory T cells (*Treg*)

The reaction rate of the regulatory T cells reads

| $\frac{dT_{reg}}{dt}=S_{Treg}+\rho_{Treg} T_{reg} \left( \frac{a}{1+c} \right)-\pi_{Treg}T_{reg}$ | (55) |
| --- | --- |

where the first term describes their production rate from the thymus. The next term is their proliferation rate due to cytokines (cytokines and anti -inflammatory). The last term describes their death rate.

- - 1. Naïve CD8^+^ T cells (*T^N^*)

The reaction rate of the naïve CD8^+^ T cells reads

| $\frac{dT^{N}}{dt}=S_{TN}-h_{T}{DC}^{*}T^{N}\left[ \left( \frac{V_{proteins}}{1+V_{proteins}} \right)+\left( \frac{As}{1+As} \right) \right]\left( \frac{c}{1+c} \right)\left( \frac{{IFN}_{\gamma}}{K_{IF}+{IFN}_{\gamma}} \right) \frac{K_{T}}{K_{T}+\left[ PD1-PDL1 \right]}-d_{TN}T^{N}$ | (56) |
| --- | --- |

where the first term describes the source term of Naïve CD8^+^ T cells, the second term describes the activation rate of naïve CD8^+^ T cells, and the last term describes the death rate of naïve CD8^+^ T cells.

- - 1. Effector CD8^+^ T cells (*T^E^*)

The reaction rate of the effector CD8+ T cells reads

| $\frac{dT^{E}}{dt}=h_{T}{DC}^{*}T^{N}\left[ \left( \frac{V_{proteins}}{1+V_{proteins}} \right)+\left( \frac{As}{1+As} \right) \right]\left( \frac{c}{1+c} \right)\left( \frac{{IFN}_{\gamma}}{K_{IF}+{IFN}_{\gamma}} \right) \frac{K_{T}}{K_{T}+\left[ PD1-PDL1 \right]}+\rho_{T}T^{E}\left( \frac{As}{1+As} \right)\left( \frac{c}{1+c} \right)\left( \frac{{IFN}_{\gamma}}{K_{IF}+{IFN}_{\gamma}} \right) \frac{K_{T}}{K_{T}+\left[ PD1-PDL1 \right]}-\pi_{TM}T^{E}-\varepsilon T^{E}\frac{\left[ PD1-PDL1 \right]}{AS}-\frac{k_{Treg_{2}}T^{E}T_{reg}}{\left( 1+T^{E} \right)\left( 1+T_{reg} \right)}-d_{TE}T^{E}$ | (57) |
| --- | --- |

where the first term describes the activation rate of the naïve CD8^+^ T cells, the second term describes the proliferation rate of the effector CD8^+^ T cells, the third term describes the conversion rate of effector CD8^+^ T cells to memory CD8^+^ T cells and the last terms describe their death rates which depend on the PD1 – PDL1 complex, the regulatory T cells, and the tumor cells.

- - 1. Macrophages

|  | (58) |
| --- | --- |

Macrophages are recruited by cytokines and IL-6 which is bound to IL-6R, die with a rate constant γa.

- - 1. Natural killer cells

The reaction rate of the natural killer cells reads

| $\frac{dNK}{dt}=\frac{\chi_{NK} c}{1+a}-\gamma_{NK}NK$ | (59) |
| --- | --- |

The first term is the production of Natural killer cells depending on the cytokines (pro/anti-inflammatory). The last term is their death rate.

- - 1. Naïve Β cells (*Β^N^*) (From Ref. [23])

(60)

where the first term describes the source term of Naïve B cells, the second term describes the activation rate of naıve B cells, and the last term describes the death rate of naıve B cells

- - 1. Activated Β cells (*Β^Α^*) (From Ref. [23])

(61)

where the first term describes the activation rate of naıve B cells, the second term describes the proliferation rate of activated B cells, the following term describes the clearance rate of activated B cells, the next term describes the differentiation rate of activated B cells into short-lived antibody-secreting plasma cells, and the last term describes the differentiation rate of activated B cells into long-lived antibody-secreting plasma cells.

- - 1. Long-lived plasma (antibody-secreting) by B cells (*P^L^*) From Ref. [23])

(62)

where the first term describes the differentiation rate of activated B cells into long-lived antibody-secreting plasma cells by cell-to-cell interactions between effector and memory CD4+ cells (Th^E^ & Th^M^) and activated B cells (B^A^), and the second term describes the death rate of long-lived antibody-secreting plasma cell

- - 1. Short-lived plasma (antibody-secreting) by B cells (*P^S^*) (From Ref. [23])

(63)

where the first term describes the differentiation rate of activated B cells into short-lived antibody-secreting plasma cells, and the last term describes the death rate of short-lived antibody-secreting plasma cells

- - 1. Memory CD4+ T cells (Th^M^)

(64)

where the first term describes the conversion rate of effector CD4+ cells to memory CD4+-cell, and the last term describes the clearance rate of memory CD4+ cells

- - 1. Memory CD8+ T cells (T^M^)

(65)

where the first term describes the conversion rate of effector CD8+ cells to memory CD8+-cell, and the last term describes the clearance rate of memory CD8+ cells

1. Cancer Cells

Cancer cell proliferation depends on the oxygen levels in the tissue, and their death rate depends on the interaction of cancer cells with immune cells (effector CD8^+^ T cells, natural killer cells, type 1 macrophages and neutrophils) as well as on the effect of cancer therapy [40, 41]. We assume that cancer cells are not infected by the virus.

The mass balance of the tumor cell density is given by a convection-diffusion-reaction equation and it reads

|  | (66) |
| --- | --- |

Where the reaction term is given by the proliferation rate which depends on the concentration of the oxygen and by the death rate which depends on the tumor cell density and the immune cells (effector CD8+, natural killer, type 1 macrophages and neutrophils).

1. Vascular density

(67)

where $S_{v}^{0}$ is the vascular density of the normal lung and ${[EC}_{o}]$ is the initial endothelial cell population.

1. Oxygen consumption rate[19]

(68)

$V_{O2}=D_{LO2}(P_{A}-P_{b})$

where $D_{LO2}$ is the lung diffusing capacity, $P_{A}$ is the partial pressure of oxygen (*P_O2_*) in alveolar air and $P_{b}$ is the mean *P_O2_* in pulmonary capillary.

The oxygen diffusion can be represented as two components in series, one associated with the alveolar membrane $D_{MO2}$ and one associated with erythrocytes $D_{eO2}$

(69a)

The membrane component is estimated as

(69b)

$$D_{MO2}=K_{O2}\frac{\left( \frac{1}{2} \right)[S\left( A \right)+S\left( c \right)]}{\tau_{hb}}$$

where $K_{O2}$ is the Krogh diffusion constant, $S\left( A \right)$ is the average of the alveolar surface area, $S\left( c \right)$ is the capillary surface area and $\tau_{hb}$ is the harmonic mean of the distance between the alveolar surface and the erythrocyte surface.

(70)

The erythrocyte component is calculated from

(71)

$$D_{eO2}=\theta_{O2}V(c)$$

where $V(c)$ is the pulmonary capillary blood volume and $\theta_{O2}$ is the oxygen unloading conductance of blood.

The partial pressure of oxygen (*P_O2_*) in pulmonary capillary calculated from

$$P_{b}=P_{A}-\frac{V_{o2}^{max}}{D_{LO2}}$$

The oxyhemoglobin saturation according to the Hill equation

(72)

(73)

- 1. Functional vascular density

To quantify the vascular density we assume that it is affected by the decrease in the vessel diameter (d/d_o_) caused by increased number of cancer cells [42] and elevation of solid stress [18] and by the permeability of the tumor vessel wall [43].

The functional vascular density will be given from:

(74)

whereas $S_{V}^{0}$ will depend on vessel wall pore size and $\rho_{v}^{EC}$the density of endothelial cells which is given below. Vessel wall pore size depend on IFNγ concentration as described below (page 7).

- 1. Oxygen Concentration

The rate of change of oxygen in tissues depends on its transport through convection and diffusion, minus the amount of oxygen consumed by cells, plus the amount that enters the tissue from the blood vessels [44, 45], i.e.,

(75)

| $\frac{\partial c_{ox}}{\partial t}+\nabla\cdot\left(c_{ox}\mathbf{v}^{f}\right)=D_{ox}\nabla^{2}c_{ox}-\frac{A_{ox}c_{ox}}{c_{ox}+k_{ox}}\Phi^{C}+P_{er}S_{V}\left(C_{iox}-c_{ox}\right)$, |
| --- |

where *c_ox_* is the oxygen concentration, *D_ox_* is the diffusion coefficient of oxygen in the interstitial space, *A_ox_* and *k_ox_* are oxygen uptake parameters, *P_er_* is the vascular permeability of oxygen that describes diffusion across the tumor vessel wall and *C_iox_* is the oxygen concentration in the vessels.

1. Vaccination-induced Immunity [46-49]

The model considers separately the mechanisms of mRNA and vector vaccines. The vaccines , as particles, either lipid nanoparticles in the case of mRNA vaccines or viral-vector in the case of vector vaccines, enter host cells and either induce DNA transcription to mRNA (vector vaccine) and then translation into viral antigen or result directly in translation of viral antigens (mRNA vaccine). Subsequently, vaccine-induced peptides exit the cells and interact with dendritic cells to produce antigen presenting cells. These subsequently activate T cells and B cells to create CD4+ and CD8+ effector and memory T cells as well as short-lived and long-lived plasma (antibody-secreting) B cells.

- - 1. Free vaccine [adenovirus (DNA) or lipid coat (mRNA)]

(76)

where the terms respectively describe convectional transport of free vaccine particles (adenovirus or lipid coat) in lung, the binding of the vaccine particles to cells (Healthy and Antigen presenting cells-APCs) membranes, the detachment of bound vaccine particles from cells membranes and the inactivation of the vaccine particles.

- - 1. Bound vaccine [adenovirus (DNA) or lipid coat (mRNA)]

(77)

where the terms respectively describe the binding/unbinding of the vaccine particles on the cells membrane, the internalization of the bound vaccine particles into the cells and the last term is the degradation of the bound vaccine particles.

- - 1. Internalized vaccine [adenovirus (DNA)]

(78)

where the first term describes the internalization of the bound vaccine particles and the second term describes the constant deradation of the internalized vaccine particles.

- - 1. DNA Transcription [adenovirus (DNA)]

(79)

$$\frac{d{Va}_{Tran}}{dt}=K_{Tran}{Va}_{int}-d_{VaTran}$$

where the first term describes the DNA transcription of the internalized vaccine particles and the second term describes the constant degradation of the translation of the DNA.

- - 1. Production Viral proteins [adenovirus (DNA)]

(80)

$$\frac{dV_{proteins}}{dt}=K_{protein}{Va}_{Tran}-d_{Vprotein}$$

where the first term describes the production of viral protein or viral antigen from the translation of the mRNA and the last describes the constant degradation of viral proteins.

- - 1. Internalized vaccine [lipid coat (mRNA)]

(81)

where the first term describes the internalization of the bound vaccine particles, the second term describes the constant degradation of the internalized vaccine particles.

- - 1. Production Viral proteins [lipid coat (mRNA)]

(82)

$$\frac{dV_{proteins}}{dt}=K_{protein}{Va}_{int}-d_{Vprotein}$$

where the first term describes the production of viral protein or viral antigen from the translation of the mRNA and the last describes the constant degradation of viral proteins.

**SI References**

1. Voutouri C, Nikmaneshi MR, Hardin CC, Patel AB, Verma A, Khandekar MJ, et al. In silico dynamics of COVID-19 phenotypes for optimizing clinical management. Proceedings of the National Academy of Sciences. 2021;118(3):e2021642118.

2. Hachmann NP, Miller J, Collier A-rY, Ventura JD, Yu J, Rowe M, et al. Neutralization escape by SARS-CoV-2 Omicron subvariants BA. 2.12. 1, BA. 4, and BA. 5. New England Journal of Medicine. 2022;387(1):86-8.

3. Qu P, Faraone J, Evans JP, Zou X, Zheng Y-M, Carlin C, et al. Neutralization of the SARS-CoV-2 omicron BA. 4/5 and BA. 2.12. 1 subvariants. New England Journal of Medicine. 2022;386(26):2526-8.

4. Tuekprakhon A, Nutalai R, Dijokaite-Guraliuc A, Zhou D, Ginn HM, Selvaraj M, et al. Antibody escape of SARS-CoV-2 Omicron BA. 4 and BA. 5 from vaccine and BA. 1 serum. Cell. 2022;185(14):2422-33. e13.

5. Wang Q, Guo Y, Iketani S, Nair MS, Li Z, Mohri H, et al. Antibody evasion by SARS-CoV-2 Omicron subvariants BA. 2.12. 1, BA. 4 and BA. 5. Nature. 2022;608(7923):603-8.

6. Beigel JH, Tomashek KM, Dodd LE. Remdesivir for the treatment of Covid-19-preliminary report. Reply. The New England journal of medicine. 2020;383(10):994-.

7. Jayk Bernal A, Gomes da Silva MM, Musungaie DB, Kovalchuk E, Gonzalez A, Delos Reyes V, et al. Molnupiravir for oral treatment of Covid-19 in nonhospitalized patients. New England Journal of Medicine. 2022;386(6):509-20.

8. Hammond J, Leister-Tebbe H, Gardner A, Abreu P, Bao W, Wisemandle W, et al. Oral nirmatrelvir for high-risk, nonhospitalized adults with Covid-19. New England Journal of Medicine. 2022;386(15):1397-408.

9. Pilvankar MR, Yong HL, Ford Versypt AN. A Glucose-Dependent Pharmacokinetic/Pharmacodynamic Model of ACE Inhibition in Kidney Cells. Processes. 2019;7(3):131.

10. Pilvankar MR, Higgins MA, Versypt ANF. Mathematical Model for Glucose Dependence of the Local Renin–Angiotensin System in Podocytes. Bulletin of mathematical biology. 2018;80(4):880-905.

11. Lo A, Beh J, De Leon H, Hallow MK, Ramakrishna R, Rodrigo M, et al. Using a systems biology approach to explore hypotheses underlying clinical diversity of the renin angiotensin system and the response to antihypertensive therapies. Clinical trial simulations: Springer; 2011. p. 457-82.

12. Mok W, Stylianopoulos T, Boucher Y, Jain RK. Mathematical modeling of herpes simplex virus distribution in solid tumors: implications for cancer gene therapy. Clinical cancer research : an official journal of the American Association for Cancer Research. 2009;15(7):2352-60. doi: 10.1158/1078-0432.CCR-08-2082. PubMed PMID: 19318482; PubMed Central PMCID: PMCPMC2872130.

13. Smith AM, McCullers JA, Adler FR. Mathematical model of a three-stage innate immune response to a pneumococcal lung infection. J Theor Biol. 2011;276(1):106-16. doi: 10.1016/j.jtbi.2011.01.052. PubMed PMID: 21300073; PubMed Central PMCID: PMCPMC3066295.

14. Dunster JL, Byrne HM, King JR. The resolution of inflammation: a mathematical model of neutrophil and macrophage interactions. Bull Math Biol. 2014;76(8):1953-80. doi: 10.1007/s11538-014-9987-x. PubMed PMID: 25053556.

15. Su Z, Wu Y. A Multiscale and Comparative Model for Receptor Binding of 2019 Novel Coronavirus and the Implication of its Life Cycle in Host Cells. bioRxiv. 2020;10.1101/2020.02.20.958272. doi: 10.1101/2020.02.20.958272. PubMed PMID: 32511419; PubMed Central PMCID: PMCPMC7268058.

16. Mahasa KJ, Eladdadi A, de Pillis L, Ouifki R. Oncolytic potency and reduced virus tumor-specificity in oncolytic virotherapy. A mathematical modelling approach. PLoS One. 2017;12(9):e0184347. doi: 10.1371/journal.pone.0184347. PubMed PMID: 28934210; PubMed Central PMCID: PMCPMC5608221.

17. !!! INVALID CITATION !!! 12.

18. Mpekris F, Angeli S, Pirentis AP, Stylianopoulos T. Stress-mediated progression of solid tumors: effect of mechanical stress on tissue oxygenation, cancer cell proliferation, and drug delivery. Biomech Model Mechanobiol. 2015;14(6):1391-402. doi: 10.1007/s10237-015-0682-0. PubMed PMID: 25968141; PubMed Central PMCID: PMCPMC4568293.

19. Weibel ER, Sapoval B, Filoche M. Design of peripheral airways for efficient gas exchange. Respir Physiol Neurobiol. 2005;148(1-2):3-21. doi: 10.1016/j.resp.2005.03.005. PubMed PMID: 15921964.

20. Roy TK, Secomb TW. Theoretical analysis of the determinants of lung oxygen diffusing capacity. J Theor Biol. 2014;351:1-8. doi: 10.1016/j.jtbi.2014.02.009. PubMed PMID: 24560722; PubMed Central PMCID: PMCPMC4070740.

21. Lai X, Friedman A. Combination therapy of cancer with cancer vaccine and immune checkpoint inhibitors: A mathematical model. PLoS One. 2017;12(5):e0178479. doi: 10.1371/journal.pone.0178479. PubMed PMID: 28542574; PubMed Central PMCID: PMCPMC5444846.

22. Zhu H, Melder RJ, Baxter LT, Jain RK. Physiologically based kinetic model of effector cell biodistribution in mammals: implications for adoptive immunotherapy. Cancer Res. 1996;56(16):3771-81. PubMed PMID: 8706023.

23. Lee HY, Topham DJ, Park SY, Hollenbaugh J, Treanor J, Mosmann TR, et al. Simulation and prediction of the adaptive immune response to influenza A virus infection. J Virol. 2009;83(14):7151-65. doi: 10.1128/JVI.00098-09. PubMed PMID: 19439465; PubMed Central PMCID: PMCPMC2704765.

24. Haghnegahdar A, Zhao J, Feng Y. Lung Aerosol Dynamics of Airborne Influenza A Virus-Laden Droplets and the Resultant Immune System Responses: An In Silico Study. J Aerosol Sci. 2019;134:34-55. doi: 10.1016/j.jaerosci.2019.04.009. PubMed PMID: 31983771; PubMed Central PMCID: PMCPMC6980466.

25. Pfizer. Pfizer announces additional phase 2/3 study results confirming robust efficacy of novel COVID-19 oral antiviral treatment candidate in reducing risk of hospitalization or death. 2021.

26. Bonam SR, Kotla NG, Bohara RA, Rochev Y, Webster TJ, Bayry J. Potential immuno-nanomedicine strategies to fight COVID-19 like pulmonary infections. Nano today. 2021;36:101051.

27. Nechipurenko YD, Semyonov DA, Lavrinenko IA, Lagutkin DA, Generalov EA, Zaitceva AY, et al. The role of acidosis in the pathogenesis of severe forms of COVID-19. Biology. 2021;10(9):852.

28. Burioni R, Topol EJ. Assessing the human immune response to SARS-CoV-2 variants. Nature Medicine. 2021;27(4):571-2.

29. Fernandes Q, Inchakalody VP, Merhi M, Mestiri S, Taib N, Moustafa Abo El-Ella D, et al. Emerging COVID-19 variants and their impact on SARS-CoV-2 diagnosis, therapeutics and vaccines. Annals of medicine. 2022;54(1):524-40.

30. Borella R, De Biasi S, Paolini A, Boraldi F, Lo Tartaro D, Mattioli M, et al. Metabolic reprograming shapes neutrophil functions in severe COVID‐19. European Journal of Immunology. 2022;52(3):484-502.

31. McKenna E, Wubben R, Isaza-Correa JM, Melo AM, Mhaonaigh AU, Conlon N, et al. Neutrophils in COVID-19: not innocent bystanders. Frontiers in Immunology. 2022;13:864387.

32. Rawat S, Vrati S, Banerjee A. Neutrophils at the crossroads of acute viral infections and severity. Molecular Aspects of Medicine. 2021;81:100996.

33. Ranard BL, Chow CC, Megjhani M, Asgari S, Park S, Vodovotz Y. A mathematical model of SARS‐CoV‐2 immunity predicts paxlovid rebound. Journal of Medical Virology. 2023;95(6):e28854.

34. Esmaeilzadeh A, Jafari D, Tahmasebi S, Elahi R, Khosh E. Immune-based therapy for COVID-19. Coronavirus Disease-COVID-19: Springer; 2021. p. 449-68.

35. Allegra A, Di Gioacchino M, Tonacci A, Musolino C, Gangemi S. Immunopathology of SARS-CoV-2 infection: immune cells and mediators, prognostic factors, and immune-therapeutic implications. International journal of molecular sciences. 2020;21(13):4782.

36. Wang Y, Perlman S. COVID-19: inflammatory profile. Annual review of medicine. 2022;73:65-80.

37. Vetter P, Eberhardt CS, Meyer B, Martinez Murillo PA, Torriani G, Pigny F, et al. Daily viral kinetics and innate and adaptive immune response assessment in COVID-19: a case series. MSphere. 2020;5(6):10.1128/msphere. 00827-20.

38. Fara A, Mitrev Z, Rosalia RA, Assas BM. Cytokine storm and COVID-19: a chronicle of pro-inflammatory cytokines. Open biology. 2020;10(9):200160.

39. Huang C-G, Dutta A, Huang C-T, Chang P-Y, Hsiao M-J, Hsieh Y-C, et al. Relative COVID-19 viral persistence and antibody kinetics. Pathogens. 2021;10(6):752.

40. Voutouri C, Kirkpatrick ND, Chung E, Mpekris F, Baish JW, Munn LL, et al. Experimental and computational analyses reveal dynamics of tumor vessel cooption and optimal treatment strategies. Proc Natl Acad Sci U S A. 2019;116(7):2662-71. doi: 10.1073/pnas.1818322116. PubMed PMID: 30700544; PubMed Central PMCID: PMCPMC6377457.

41. Mpekris F, Voutouri C, Baish JW, Duda DG, Munn LL, Stylianopoulos T, et al. Combining microenvironment normalization strategies to improve cancer immunotherapy. Proc Natl Acad Sci U S A. 2020;117(7):3728-37. doi: 10.1073/pnas.1919764117. PubMed PMID: 32015113; PubMed Central PMCID: PMCPMC7035612.

42. Griffon-Etienne G, Boucher Y, Brekken C, Suit HD, Jain RK. Taxane-induced apoptosis decompresses blood vessels and lowers interstitial fluid pressure in solid tumors: clinical implications. Cancer Res. 1999;59(15):3776-82. PubMed PMID: 10446995.

43. Stylianopoulos T, Jain RK. Combining two strategies to improve perfusion and drug delivery in solid tumors. Proc Natl Acad Sci U S A. 2013;110(46):18632-7. doi: 10.1073/pnas.1318415110. PubMed PMID: 24167277; PubMed Central PMCID: PMCPMC3832007.

44. Kim Y, Stolarska MA, Othmer HG. The role of the microenvironment in tumor growth and invasion. Prog Biophys Mol Biol. 2011;106(2):353-79. doi: 10.1016/j.pbiomolbio.2011.06.006. PubMed PMID: 21736894; PubMed Central PMCID: PMCPMC3156881.

45. Roose T, Netti PA, Munn LL, Boucher Y, Jain RK. Solid stress generated by spheroid growth estimated using a linear poroelasticity model☆. Microvasc Res. 2003;66(3):204-12. doi: 10.1016/s0026-2862(03)00057-8. PubMed PMID: ISI:000186649000005.

46. Li Y, Tenchov R, Smoot J, Liu C, Watkins S, Zhou Q. A Comprehensive Review of the Global Efforts on COVID-19 Vaccine Development. ACS Cent Sci. 2021;7(4):512-33. doi: 10.1021/acscentsci.1c00120. PubMed PMID: 34056083; PubMed Central PMCID: PMCPMC8029445.

47. Dong Y, Dai T, Wei Y, Zhang L, Zheng M, Zhou F. A systematic review of SARS-CoV-2 vaccine candidates. Signal Transduct Target Ther. 2020;5(1):237. doi: 10.1038/s41392-020-00352-y. PubMed PMID: 33051445; PubMed Central PMCID: PMCPMC7551521.

48. Callaway E. The race for coronavirus vaccines: a graphical guide. Nature. 2020;580(7805):576-7. doi: 10.1038/d41586-020-01221-y. PubMed PMID: 32346146.

49. Pushparajah D, Jimenez S, Wong S, Alattas H, Nafissi N, Slavcev RA. Advances in gene-based vaccine platforms to address the COVID-19 pandemic. Adv Drug Deliv Rev. 2021;170:113-41. doi: 10.1016/j.addr.2021.01.003. PubMed PMID: 33422546; PubMed Central PMCID: PMCPMC7789827.
